# Functional assignment of KEOPS/EKC complex subunits in the biosynthesis of the universal t^6^A tRNA modification

**DOI:** 10.1093/nar/gkt720

**Published:** 2013-08-14

**Authors:** Ludovic Perrochia, Dorian Guetta, Arnaud Hecker, Patrick Forterre, Tamara Basta

**Affiliations:** ^1^Institut de Génétique et Microbiologie, Université Paris-Sud, IFR115, UMR8621-CNRS, 91405 Orsay, France and ^2^Université de Lorraine, UMR 1136 INRA/Université de Lorraine Interactions Arbres-Microorganismes, Labex ARBRE, FR EFABA, Faculté des Sciences, 54500 Vandoeuvre, France

## Abstract

N^6^-threonylcarbamoyladenosine (t^6^A) is a universal tRNA modification essential for normal cell growth and accurate translation. In Archaea and Eukarya, the universal protein Sua5 and the conserved KEOPS/EKC complex together catalyze t^6^A biosynthesis. The KEOPS/EKC complex is composed of Kae1, a universal metalloprotein belonging to the ASHKA superfamily of ATPases; Bud32, an atypical protein kinase and two small proteins, Cgi121 and Pcc1. In this study, we investigated the requirement and functional role of KEOPS/EKC subunits for biosynthesis of t^6^A. We demonstrated that Pcc1, Kae1 and Bud32 form a minimal functional unit, whereas Cgi121 acts as an allosteric regulator. We confirmed that Pcc1 promotes dimerization of the KEOPS/EKC complex and uncovered that together with Kae1, it forms the tRNA binding core of the complex. Kae1 binds l-threonyl-carbamoyl-AMP intermediate in a metal-dependent fashion and transfers the l-threonyl-carbamoyl moiety to substrate tRNA. Surprisingly, we found that Bud32 is regulated by Kae1 and does not function as a protein kinase but as a P-loop ATPase possibly involved in tRNA dissociation. Overall, our data support a mechanistic model in which the final step in the biosynthesis of t^6^A relies on a strictly catalytic component, Kae1, and three partner proteins necessary for dimerization, tRNA binding and regulation.

## INTRODUCTION

**Figure 1.**
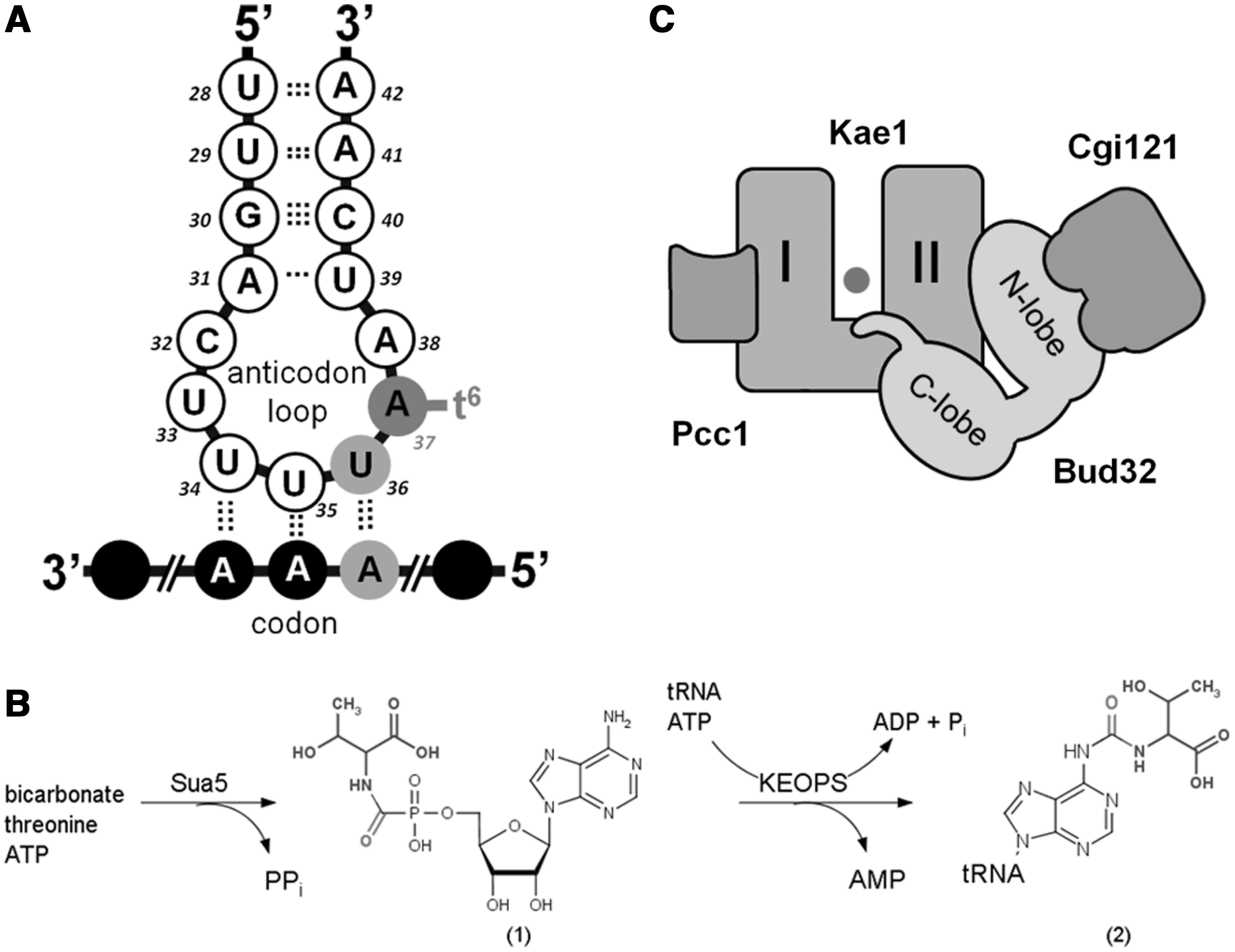
Biosynthesis of t^6^A modification in Archaea and Eukarya. (**A**) *Schematic representation of the anticodon loop of tRNA^Lys^ from E. coli*. N^6^-threonylcarbamoyladenosine (t^6^A) at the position 37 is indicated with full circle in dark gray and the major determinant nucleoside U36 is indicated in gray. Messenger RNA is indicated in black circles and the interaction of the anticodon with the codon triplet AAA is indicated by dotted lines. t^6^A stabilizes the anticodon loop structure by preventing intraloop bonding between U_33_ and A_37_. (**B**) *Reaction scheme leading to t^6^A biosynthesis in Archaea and Eukarya*. Sua5 catalyzes the formation of the l-threonyl-carbamoyl-AMP (TC-AMP) intermediate from bicarbonate, threonine and ATP. KEOPS catalyzes the final step of the reaction in which l-threonyl-carbamoyl moiety is transferred from TC-AMP to A37 of substrate tRNA. tRNA binding to KEOPS significantly stimulates hydrolysis of ATP by KEOPS. (**C**) *Schematic representation of the architecture of the KEOPS complex*. The arrangement of different subunits is represented according to the reported composite model structure (2). For Kae1, two domains of the ASHKA fold are indicated by I and II and the iron atom in the active site cavity is represented as a full circle.

Transfer RNA molecules carry a variety of posttranscriptionally modified nucleotides derived from the canonical A, C, G and U residues, and their formation is catalyzed by numerous specific enzymes. Specifically, anticodon-domain modifications enhance the accuracy of codon binding, maintain the translational reading frame and enable translocation of the tRNA from the A-site to the P-site of the ribosome [reviewed in (1)]. Among those, N^6^-threonylcarbamoyl adenosine (t^6^A) is a modified nucleotide found exclusively at the position 37, adjacent to the anticodon, of all tRNAs that decode ANN codons (where N is any of the four canonical nucleotides A, T, G or C) (Figure 1A) (3–5). This modification is found in all cellular organisms and also in mitochondria and plastids, but it is absent only in obligate endosymbiotes with highly reduced genomes, such as *Carsonella ruddii* (5–7). t^6^A modification is essential for normal cell growth and accurate translation by the ribosome. It stabilizes the anticodon loop structure by preventing intraloop hydrogen bonding, and it facilitates anticodon–codon pairing by mediating base-stacking interaction at the ribosomal decoding site, thus preventing frameshifting in the course of translation (8–11). Recently, a cyclic form of t^6^A (ct^6^A), an active ester with an oxazolone ring, was detected in several organisms, including *Escherichia coli*, *Saccharomyces cerevisiae* and spinach, suggesting that this is the native form for this modification in some populations of bacteria, fungi and plants (12).

Recently, it was shown that the biosynthesis of t^6^A-modified tRNA in bacteria is a complex reaction that requires four proteins, YrdC (ortholog of Sua5), YgjD, YeaZ and YjeE (14–17). Of those, YrdC/Sua5 proteins are nearly ubiquitous, YgjD proteins and their homologs belong to the small set of ∼60 universal proteins present in all cellular life forms and YeaZ and YjeE are specific for the bacterial domain (18,19). YrdC was shown to catalyze the formation of l-threonyl-carbamoyl adenylate intermediate (TC-AMP) from threonine, bicarbonate and ATP (17). YgjD and YeaZ form a stable heterodimeric complex in solution, whereas YjeE binds to YgjD–YeaZ only in the presence of ATP or ADP (20,21). In the presence of TC-AMP, the ternary YgjD–YeaZ–YjeE complex catalyzes the last step of the reaction, i.e. transfer of l-threonyl-carbamoyl to A37, and this reaction does not require the addition of ATP (17).

In Archaea and Eukarya, Sua5 (YrdC homolog) and the KEOPS/EKC complex together catalyze the biosynthesis of t^6^A modification in an ATP-dependent fashion from threonine, bicarbonate and substrate tRNA (13,23). Sua5 was shown to catalyze the hydrolysis of ATP to AMP and pyrophosphate in a threonine-dependent manner, whereas KEOPS binds tightly to tRNA and converts ATP to ADP and inorganic phosphate (Figure 1B). KEOPS/EKC complex, here forth called KEOPS for simplicity, was initially discovered in *S. cerevisiae* using genetic and transcriptomic studies, which indicated that KEOPS functions in telomere maintenance and transcription regulation (24,25). KEOPS stands for ‘Kinase Endopeptidase and Other proteins of Small size’ and indicates that the complex consists of a protein kinase Bud32, a putative endopeptidase Kae1 (homolog of YgjD) and three uncharacterized proteins of small size, Pcc1, Cgi121 and Gon7 (24). KEOPS complex proteins are conserved throughout archaeal and eukaryal domains, including humans, except for Gon7, which is found only in fungi (2,24,25).

Kae1 was initially annotated as endopeptidase because its bacterial homolog YgjD was erroneously reported to possess an O-sialoglycoprotein protease activity (26). It was shown later on that Kae1 is an iron metalloprotein with a low ATPase activity (19,27).

Bud32, also called PRPK (p53-related protein kinase), is an atypical serine/threonine kinase that received much attention in the past because it was shown to interact with and phosphorylate the human oncosuppressor p53 protein *in vitro* and *in vivo* when expressed in human cell lines (35,36). Intriguingly, Bud32 lacks the conventional structural elements necessary for the substrate recognition as well as a lysyl residue that in all other Ser/Thr kinases, participates in the catalytic event by interacting with the transferred ATP γ-phosphate (2,30). Bud32 autophosphorylation activity is stimulated *in vitro* by Cgi121, which lacks recognizable structural domains, thus precluding any functional predictions (2,27).

Three-dimensional structures were resolved for several subcomplexes and individual proteins of KEOPS, which allowed construction of a model for the entire complex (2,27). The model structure has a linear architecture where Cgi121 binds to Bud32, which binds to Kae1, which in turn binds to Pcc1 (2) (Figure 1C). The KEOPS complex forms homodimers in solution, and Pcc1 was proposed to function as a dimerization module (2,23). Closest structural counterpart of Pcc1 is K homology (KH) domain, which functions as a single-stranded DNA and an RNA binder (2).

Eukaryotes harbor two chromosomally encoded orthologs belonging to the Kae1/YgjD family: Kae1, which localizes in the cytoplasm, and a second version, dubbed Qri7 in *S. cerevisiae*, which was shown to localize in the mitochondria (32,33). Wen and colleagues recently showed that Sua5 and Qri7 from *S. cerevisiae* together catalyze the biosynthesis of t^6^A in the presence of threonine, ATP and substrate tRNA (34). Qri7 was shown to catalyze by itself the last step of the reaction in presence of TC-AMP, ATP and substrate tRNA. Qri7 can therefore function without the additional partners present in KEOPS, raising the question of the requirement and functional role of these partner proteins in the biosynthesis of t^6^A (34).

In the present study, using KEOPS from the archaeon *Pyrococcus abyssi* (PaKEOPS) as a model system, we determined a minimal set of KEOPS proteins required for the biosynthesis of t^6^A, and we propose a distinct function for each of the four proteins. Our data support a mechanistic model in which Kae1 is the genuine catalytic component of the complex that operates together with three partner proteins that have distinct functions in tRNA binding, dimerization and allosteric regulation.

## MATERIALS AND METHODS

### Cloning procedures

The cloning of genes encoding Sua5, Kae1 and the KEOPS complex of *P. abyssi* in expression vectors was described previously (23).

Three different strategies were used for cloning of genes encoding binary and ternary subcomplexes and individual KEOPS proteins: (i) polymerase chain reaction amplification followed by cloning into an expression vector, (ii) restriction digestion of plasmids and subcloning of genes into expression vectors and (iii) chemical gene synthesis followed by cloning into expression vectors. The procedures used for each construct are listed in Supplementary Table S1. All oligonucleotide primers are available on request.

### Site-directed mutagenesis

Mutant versions of KEOPS proteins were generated using the QuickChange Lightning kit (Agilent), following the protocol supplied by the manufacturer. The oligonucleotide primers were designed with the QuickChange Primer Design Program (Agilent). The primers are listed in Supplementary Table S2.

### Recombinant proteins expression and purification

Recombinant expression plasmids (Supplementary Table S1) were used for the transformation of Rosetta2 (DE3) pLysS *E. coli* expression strain (Novagen). Protein overexpression was induced by diluting a preculture in 1 l of OverNight Express Instant TB medium (Novagen) supplemented with 10% glycerol and appropriate antibiotics, and incubated overnight at 37°C and 180 rpm.

The cells were collected by centrifugation and were resuspended in lysis buffer (LBf) (see supplementary materials and methods) and sonicated on ice. Cell extract was then centrifuged at 10 000 *g* for 15 min and then heated at 65°C for 15 min, and centrifuged again at 10 000 *g* for 15 min to remove precipitated proteins. His-tagged proteins from the soluble fraction were purified by gravity-flow chromatography on a nickel nitrilotriacetate agarose resin (Ni-NTA) column (Qiagen) according to the manufacturer’s recommendations. Fractions containing recombinant proteins were pooled, concentrated to a volume of 2 ml and injected on a Superdex™200 column (GE Healthcare) equilibrated with storage buffer (LBs). The eluted fractions were analyzed by sodium dodecyl sulfate polyacrylamide gel electrophoresis (SDS-PAGE). Fractions containing pure proteins were pooled, frozen in liquid nitrogen and stored at −80°C.

Protein concentration was determined either using the Bio-Rad Protein Assay (Bio-Rad) and bovine serum albumin as standard or by measuring the absorbance at 280 nm using theoretical molar absorption coefficients. Absorption spectrum of purified proteins was measured from 1000 nm to 200 nm using UV-visible Spectrophotometer Cary-50 Scan (Varian).

### Gel filtration analysis of KEOPS protein interactions

Different combinations of proteins (100 µg each) were mixed in LBs in 200 µl of final volume and incubated for 20 min at 4°C or room temperature. The mixture was subsequently loaded on Superdex 200 10/300 (GE Healthcare) gel filtration column equilibrated with LBs. Eluted proteins were analyzed by SDS-PAGE.

### tRNA production and purification

The procedures used for tRNA production by overexpression of recombinant tRNA genes in *E. coli* were described earlier (23).

Briefly, the tRNA genes were expressed in XL1 Blue *E. coli* strain (Agilent Technologies) under the control of the T5 promoter and *lac* operator. Total tRNA was extracted from cells using Trizol (Sigma), following the manufacturer’s protocol.

For purification, RNAs were loaded on 8M urea, 12% polyacrylamide gel, and the dominant band corresponding to overexpressed tRNA was cut out. tRNA was eluted from gel in elution buffer (EB), precipitated and resuspended in water. To favor correct folding of tRNAs, the solution was heated at 70°C, cooled slowly to room temperature and stored at −80°C.

### ATP hydrolysis assay

The ATP hydrolysis reactions were carried out as described previously (23). Two micromolar of individual proteins or subcomplexes were mixed in t^6^A reaction buffer (RB) supplemented with 100 μM of cold ATP and 1 μCi of [α^32^-P] ATP (Perkins Elmer). When indicated, tRNA was added to the reaction.

Reaction was performed at 50°C for 30 min, then stopped on ice and 1 μl of the mix was spotted on 20 × 20-cm PEI–cellulose plates (Merck), pre-run in distilled water. Radioactive nucleotides were separated by thin layer chromatography (TLC) using as solvent 0.5 M KH_2_PO_3_^-^ pH 3.5 or 0.5 M LiCl, 1 M formic acid. Plates were dried and radioactivity was revealed by phosphorimaging.

### *In vitro* assay for the synthesis of t^6^A-modified tRNA

The reaction was performed in a final volume of 50 μl containing different KEOPS complex proteins at 1 µM (or other concentration when indicated), tRNA (2 μM) and 18.2 μM 14C-l-threonine (0.05 μCi, 55 Ci/mol, Hartmann Analytic) in RB. After 30 min incubation at 50°C (or other incubation times when indicated), macromolecules were precipitated by addition of 1 ml of trichloroacetic acid 15% (TCA), and incubated at 4°C for 1 h. Precipitated material was applied on pre-wet glass microfiber filters GF/F (Whatmann) using vacuum apparatus (Millipore). Filters were washed with 2 ml of 5% TCA and 3 mL of 95% EtOH and dried. Radioactivity was recorded as average counts per minute (CPM) for 2 min, with Packard Liquid Scintillation Analyzer. The amount of incorporated threonine was calculated from a standard curve obtained with different concentrations of ^14^C-l-threonine spotted on filters (1 pmol = 102.8 CPM).

### Protein phosphorylation assay

The phosphotransferase activity of Bud32 was assayed by mixing 1.5 µM of KEOPS or Bud32–Cgi121 complexes with 3 µM of Sua5 and 12 µM of tRNA (when indicated) in t^6^A RB supplemented with 25 µM of cold ATP and [y-^32^P]ATP (1 µCi per reaction, specific activity of 6000 Ci/mmol, Perkins Elmer). Incubations were performed in a final volume of 20 µl at 55°C for 30 min. After incubation, reactions were stopped on ice, and 1 µl of the reaction was analyzed on PEI–cellulose TLC. The rest of the reaction mixture was analyzed by SDS-PAGE. The gels were soaked in 16% (v/v) TCA for 10 min at 90°C and neutralized in TBE buffer. The treated gels were finally stained with InstantBlue^TM^ (Expedeon) and radiolabeled proteins visualized by phosphorimaging.

### Electrophoretic mobility shift assay

The binding reactions were carried out in a final volume of 20 μl containing 0.05 pmol (10 nM) of radioactively labeled tRNA probes and purified recombinant KEOPS complex proteins (0.5–2 μM) in binding buffer (BB). Incubations were performed for 15 min at 4°C, the products were loaded onto a 10% non-denaturating acrylamide gel and electrophoresis was performed in 1X TGE buffer at 4°C for 4 h at 7.5 V/cm. The RNA–protein complexes were visualized by autoradiography and phosphorimaging.

### Molecular docking of TC-AMP into the active site of PaKae1

Molecular docking was performed as blind docking by using the Autodock Vina 1.1.2 software (35) from the MGLTools 1.5.6 package (Molecular Graphics Laboratory of The Scripps Research Institute). This method refers to the use of a grid box that is large enough to encompass any possible ligand-receptor.

The PDB file 2IVN related to Pa-Kae1 (19) devoid of its AMP-PNP ligand and TC-AMP substrate were used, respectively, as receptor and ligand for docking. A rectangular cuboid grid box with dimensions of 15 × 10 × 15 Å, along the x, y and z axes, respectively, was defined around the active site of Pa-Kae1 to circumscribe it entirely, and to accommodate free motion of ligands.

All rotatable bonds within the ligand were allowed to rotate freely and the receptor was considered rigid. Autodock Tools 1.5.6 (36) was used to convert both ligand and receptor molecules to the right file format for docking in AutoDock Vina. Docking was performed using an ‘exhaustiveness’ value of 50 and other parameters set as default. The 10 highest-scoring docking conformations have been analyzed and visualized with the PyMOL Molecular Graphics System (Schrödinger, LLC.). The conformation presenting the best binding affinity was selected.

## RESULTS

### Purification of individual PaKEOPS proteins and subcomplexes

To study the function of each subunit of PaKEOPS, we first attempted to overexpress and purify each protein individually. Kae1 was previously purified as a monomer in solution that had pink color due to the presence of iron in the active site (19). Cgi121 could also be purified in the soluble monomeric form, but Bud32 and Pcc1 formed precipitates in the course of purification in all tested conditions (see Materials and Methods). This suggested that the latter proteins were stabilized by their partners in the PaKEOPS complex. According to the structural model of KEOPS, Pcc1 interacts with Kae1, Kae1 with Bud32 and Bud32 with Cgi121, indicating that a precise number of binary (three) and ternary (two) complexes could be produced (2). We thus overexpressed soluble recombinant Bud32 and Pcc1 as binary complexes Bud32–Cgi121, Pcc1–Kae1 and Bud32–Kae1 and ternary complex Kae1–Bud32–Cgi121 (Supplementary Figure S1). Surprisingly, we failed to obtain soluble and stable form of the second ternary complex, Pcc1–Kae1–Bud32, although we tried several different cloning strategies (see supplementary materials and methods).

Purified binary complexes all eluted as a single peak in gel filtration experiments (not shown) with apparent molecular masses corresponding to monomeric forms. The only exception was the complex Pcc1–Kae1, which eluted at a volume corresponding to higher molecular mass than expected for a monomer (∼66 kDa instead of predicted 45 kDa for monomer), suggesting that it formed a dimer in solution just like the whole PaKEOPS complex (Supplementary Figure S2). The ternary complex Kae1–Bud32–Cgi121 was purified as a single peak; however, gel filtration analyses of samples after storage showed the appearance of an additional peak corresponding to Bud32–Cgi121, indicating a spontaneous dissociation of the ternary complex. Notably, the apparent molecular mass of the ternary complex corresponded to a monomeric form, indicating that Pcc1 is required for the dimerization of PaKEOPS.

As the whole PaKEOPS complex co-purifies with tRNA of the *E. coli* expression strain (23), we analyzed all of the purified proteins and complexes including the precipitates of Pcc1 and Bud32 for the presence of tRNA. None of the subcomplexes or individual proteins presented absorbance peak at 260 nm or carried detectable amounts of tRNA, indicating that they did not form stable complexes with tRNA (data not shown).

### Pcc1, Kae1 and Bud32 are the minimal protein set required and sufficient for t^6^A formation *in vitro*

**Figure 2.**
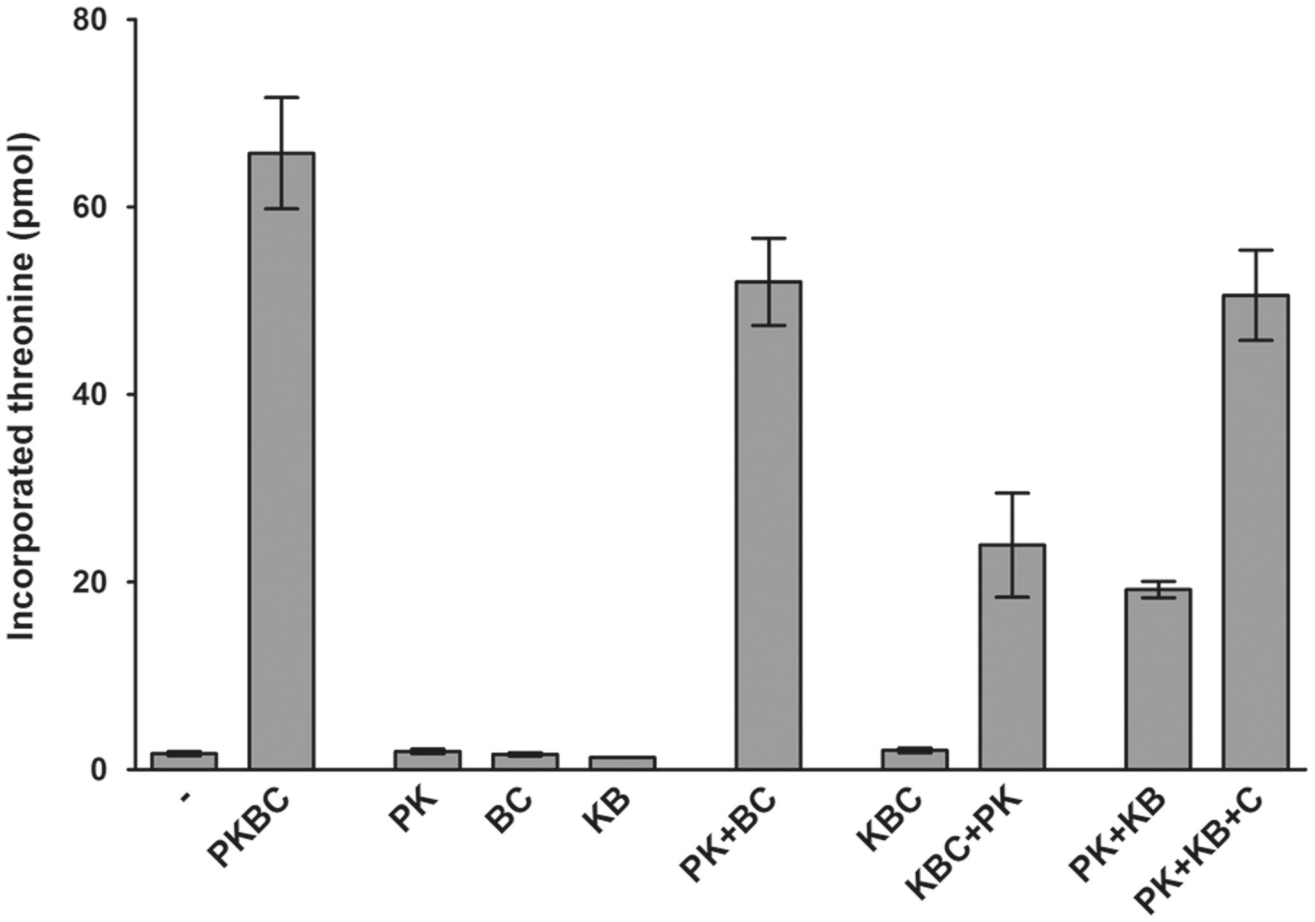
*In vitro* biosynthesis of t^6^A-modified tRNA by subcomplexes of KEOPS from *P. abyssi*. Different subcomplexes and their combinations were incubated with Sua5 from *P. abyssi*, ATP, bicarbonate, C^14^threonine and substrate tRNA (see Materials and Methods). The radioactive l-threonine incorporated in TCA-precipitated material was counted by scintillation. Error bars correspond to standard deviation from three independent experiments. Single letter designations are as follows: P, Pcc1; K, Kae1; B, Bud32 and C, Cgi121.

We next tested the t^6^A activity of different individual subcomplexes and their combinations to identify the proteins that are required for the reaction to take place. The assay mixture we used contained PaSua5, ATP, magnesium, manganese, bicarbonate, C^14^-l-threonine and substrate tRNA. The assay measures the incorporation of radioactive threonine into TCA-precipitated material, which corresponds to the formation of t^6^A-modified tRNA (23). As shown in Figure 2, the three individual binary complexes did not present detectable t^6^A synthetic activity, nor did the ternary complex Kae1–Bud32–Cgi121. Mixing equimolar amounts of Pcc1–Kae1 and Bud32–Cgi121 resulted in ∼80% of activity as compared with PaKEOPS, suggesting that these binary complexes reconstitute the whole functional PaKEOPS complex. Similarly, adding Pcc1–Kae1 to the ternary complex Kae1–Bud32–Cgi121 restored ∼36% of the activity, and suggests that Pcc1 is absolutely required for the *in vitro* synthesis of t^6^A. Finally, we measured significant amount of t^6^A formation (∼30% of activity relative to whole PaKEOPS complex) when mixing Pcc1–Kae1 and Kae1–Bud32 binary complexes. Thus, Pcc1, Kae1 and Bud32 are the minimal set of proteins required for the synthesis of t^6^A modification. The addition of Cgi121 to the mix of Pcc1–Kae1 and Kae1–Bud32 resulted in a signal comparable with the one obtained with the whole PaKEOPS complex, confirming that Cgi121 is required for the maximal activity.

We next checked if the measure of t^6^A activity corresponded to the formation of a stable PaKEOPS complex or ternary complex Pcc1–Kae1–Bud32 (Supplementary Figure S3). Using gel filtration experiments, we could confirm that the 1:1 mixture of Pcc1–Kae1 and Bud32–Cgi121 binary complexes formed a single peak corresponding to the PaKEOPS complex. When the Kae1–Bud32–Cgi121 ternary complex was combined with Pcc1–Kae1, two peaks were observed: one corresponding to PaKEOPS and one corresponding to the ternary complex Kae1–Bud32–Cgi121. This indicated that only a part of the ternary complex was used for the formation of the whole active complex, which could explain rather low t^6^A activity measured for this combination of complexes (36%, see earlier text). Interestingly, we could not detect a peak corresponding to a stable Pcc1–Kae1–Bud32 complex when mixing Pcc1–Kae1 and Kae1–Bud32 under the conditions used in t^6^A assay, although this combination of binary complexes could catalyze the formation of t^6^A. The analysis of this mixture of binary complexes to which Cgi121 was added in excess showed a peak corresponding to the whole PaKEOPS complex and an additional peak corresponding to Cgi121.

### Bud32 is responsible for the ATPase activity of PaKEOPS and this activity is required for t^6^A formation

We previously reported that PaKEOPS hydrolyzes ATP to ADP and that this ATPase activity is strongly stimulated in presence of tRNA (23). To identify the protein(s) responsible for this ATPase activity, we performed ATPase assays in presence or absence of tRNA using radioactive alpha P^32^–ATP and TLC to separate the products from unreacted ATP.

**Figure 3.**
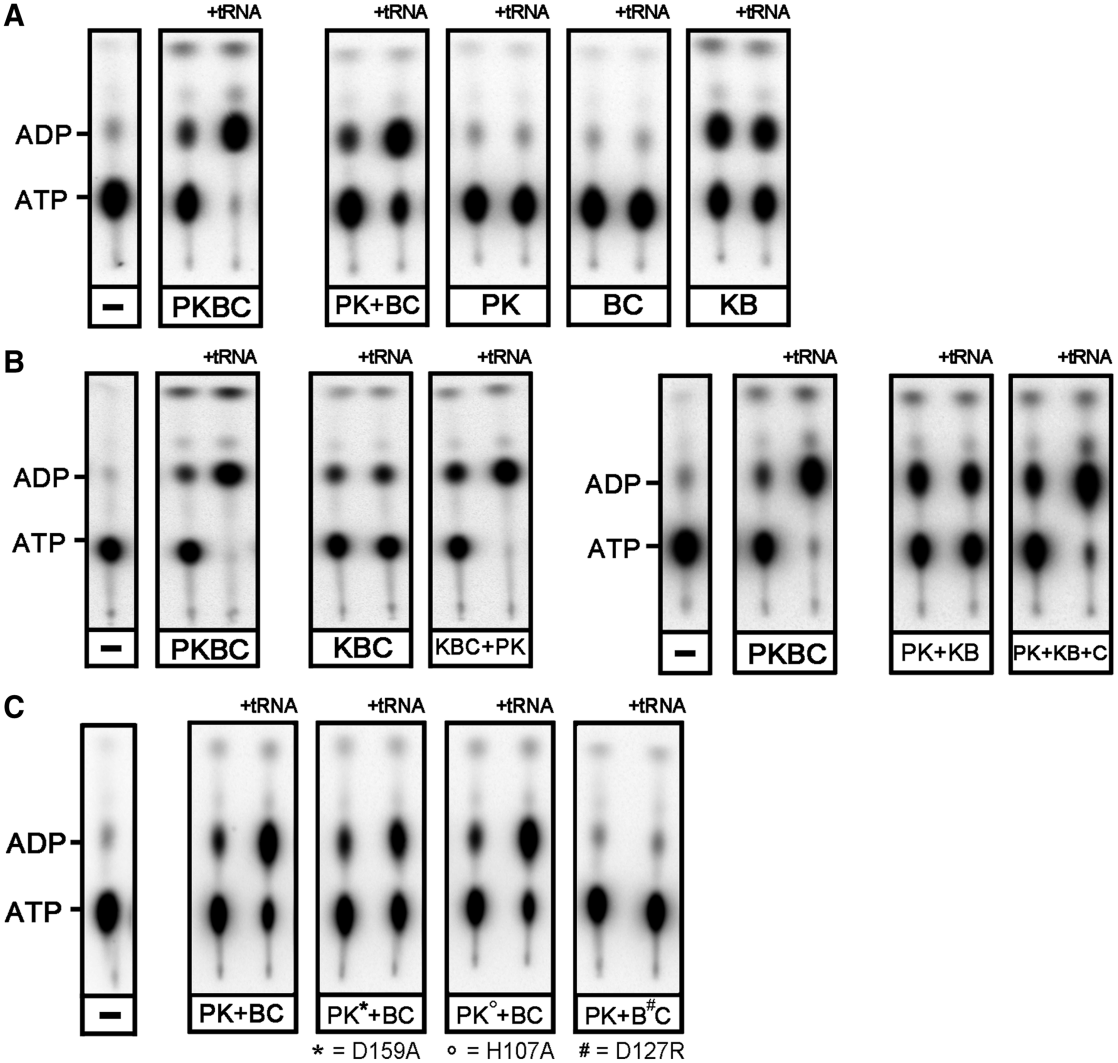
ATPase activity of wild-type and mutant KEOPS proteins from *P. abyssi*. KEOPS subcomplexes were incubated with α-P^32^ ATP in reaction buffer either alone or in combination with other proteins. Produced radiolabeled (α-P^32^)-ADP was separated by TLC and visualized by phosphorimaging. When indicated, Ec_tRNALys produced in overexpressing *E. coli* strain was added to the reaction mixture. In negative controls, indicated by a minus sign, the proteins were omitted in the reaction mixture. (**A**): ATPase activity of binary complexes. (**B**): ATPase activity of ternary complex Kae1–Bud32–Cgi121. (**C**): The ATPase activity was measured for reconstituted KEOPS complexes containing point mutations in Kae1 and Bud32 indicated at the bottom. Single letter designations are as follows: P, Pcc1; K, Kae1; B, Bud32 and C, Cgi121.

As shown in Figure 3A, binary complex Kae1–Bud32 exhibited a significant ATPase activity in the presence or absence of tRNA, whereas the two other binary complexes Pcc1–Kae1 and Bud32–Cgi121 did not. This indicated that Kae1 and/or Bud32 were responsible for the ATPase activity of PaKEOPS, but only when the two proteins interact with each other. Interestingly, whereas the ATPase activity of the whole PaKEOPS complex is stimulated by the presence of tRNA, the ATPase activity of the Kae1–Bud32 complex is independent of tRNA, suggesting that Pcc1 and/or Cgi121 were required for the response to tRNA. To test this hypothesis, we compared the ATPase activity of Kae1–Bud32–Cgi121 complex with that of the mixture of the Pcc1–Kae1 and Kae1–Bud32 binary complexes (Figure 3B). In both cases, we recorded a significant ATPase activity that was independent of tRNA. However, when Kae1–Bud32–Cgi121 was combined with Pcc1–Kae1, or Cgi121 was added to the Pcc1–Kae1 and Kae1–Bud32 assay, we observed the wild-type tRNA-stimulated ATPase activity. Taken together, these data indicate that the simultaneous presence of both Pcc1 and Cgi121 is required for the tRNA-stimulated ATPase activity of PaKEOPS.

Both Kae1 and Bud32 were described to possess an ATPase activity, and structural analyses showed that Kae1 co-crystallized with AMP-PNP (an analog of ATP) bound in the active site (19,30). To assign the ATPase activity to Kae1 or Bud32, we produced point mutation variants of Pcc1–Kae1 and Bud32–Cgi121 binary complexes in which the strictly conserved residues in the active site of Kae1 and Bud32 were mutated into arginine. For Kae1, we targeted D159, which, according to the crystal structure, forms two hydrogen bonds with the ribose of ATP (26). In the case of Bud32, we targeted a catalytic cleft residue D127, which was previously shown to result in loss of protein kinase activity (2,30).

**Figure 4.**
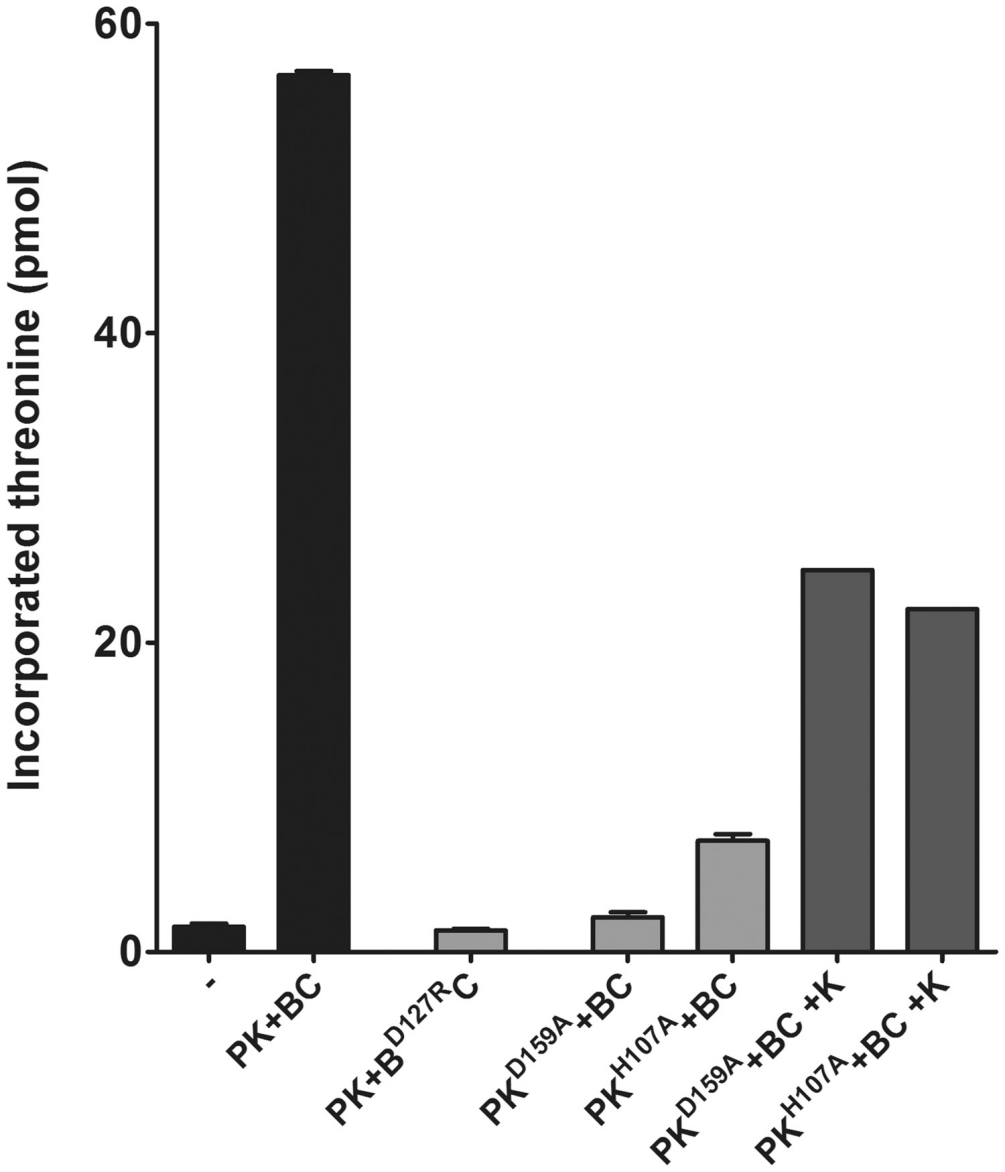
Impact of point mutations of Kae1 and Bud32 on the *in vitro* biosynthesis of t^6^A modification by KEOPS from *P. abyssi*. Whole KEOPS complexes were reconstituted by mixing two binary subcomplexes carrying point mutations. The reconstituted complexes were incubated with Sua5 from *P. abyssi*, ATP, bicarbonate, C^14^ l-threonine and substrate tRNA. The reaction mixtures were precipitated with TCA, and the radioactivity levels in the precipitates were measured by scintillation counting. The counts were converted into amount of incorporated C^14^ l-threonine into tRNA using a standard calibration curve. The negative control, which is designated with a minus sign, corresponds to the total assay mixture without proteins.

Next, whole PaKEOPS complexes that contained either Kae1^D159A^ or Bud32^D127R^ were reconstituted and their ATPase activity was measured in presence or absence of tRNA. We found that PaKEOPS (Kae1^D159A^) behaved in the same manner as the wild-type PaKEOPS, whereas PaKEOPS (Bud32^D127R^) totally lost the ATPase activity (Figure 3C). This indicated that Bud32 is the subunit responsible for the ATPase activity of PaKEOPS. Moreover, PaKEOPS (Bud32^D127R^) was inactive when assayed for the t^6^A synthetic activity, indicating that the ATPase activity of Bud32 is most likely required for the biosynthesis of t^6^A (Figure 4).

### Kae1 modifies the phosphotransferase activity of Bud32

**Figure 5.**
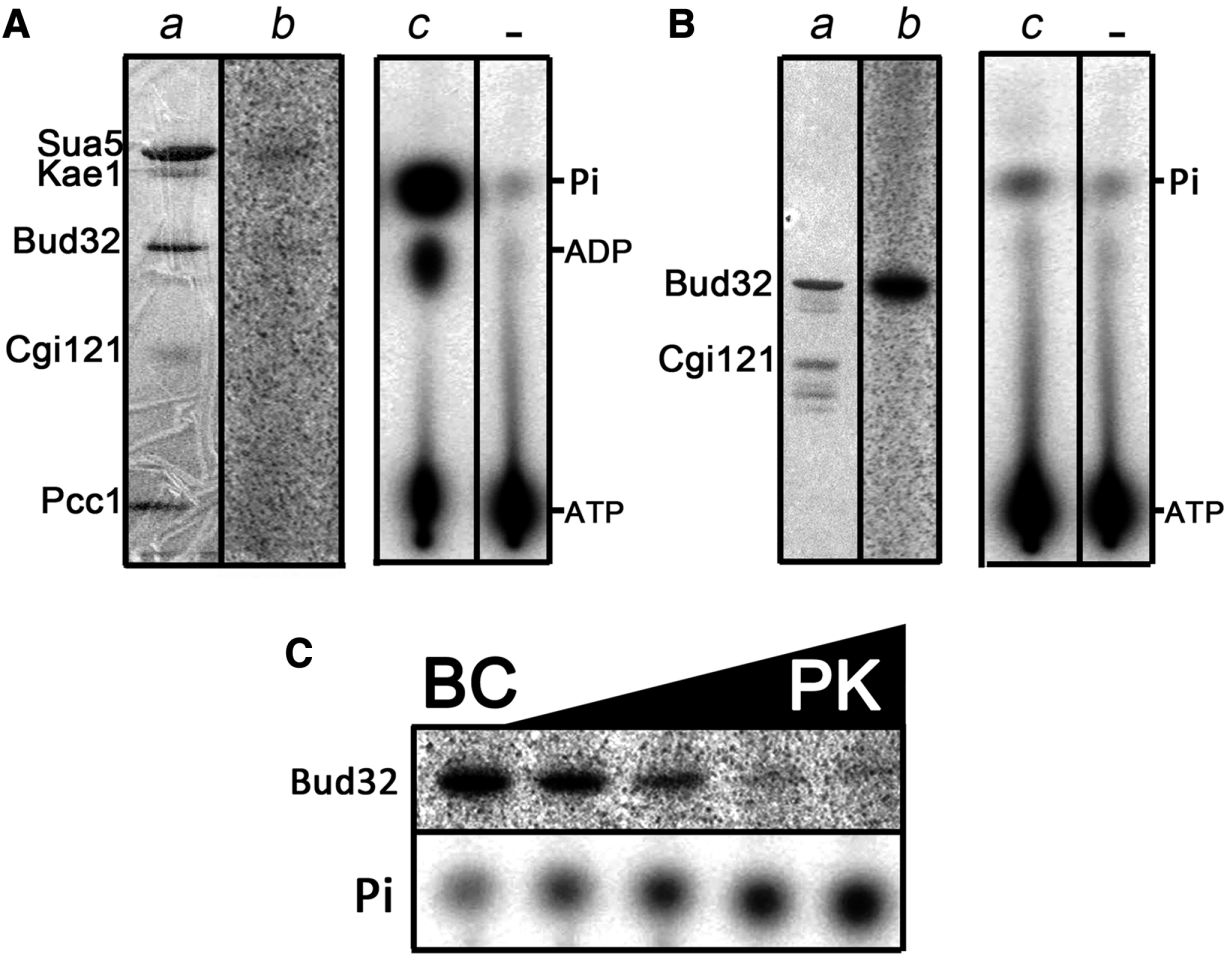
Analysis of ATPase and protein kinase activity of Bud32. (**A**) *As a part of the KEOPS complex, Bud32 does not exhibit a significant protein kinase activity.* Sua5 and the KEOPS complex were incubated under standard t^6^A assay conditions in presence of γ-P^32^ ATP (see Materials and Methods). The reaction mixture was analyzed by SDS-PAGE (part a), and the radioactivity retained by the proteins was recorded by phosphorimaging (part b). The same reaction mixture (part c) and a negative control lacking proteins (part -) was analyzed by TLC. The radioactive spots correspond to different nucleotides and free inorganic phosphate, as indicated. (**B**) *Bud32 exhibits autophosphorylation activity in presence of Cgi121.* The Bud32–Cgi121 binary complex was incubated in presence of γ-P^32^ ATP under standard t^6^A assay conditions, except that tRNA was omitted in the assay. The reaction mixture was analyzed by SDS-PAGE (part a), and the radioactivity retained by the proteins was recorded by phosphorimaging (part b). An aliquot of the reaction mixture (part c) and a negative control lacking proteins (part -) was analyzed by TLC. (**C**) *Bud32 phosphotransferase activity is switched off in presence of Kae1*. The Bud32–Cgi121 complex was incubated in presence of γ-P^32^ ATP under standard t^6^A assay conditions, except that tRNA was omitted in the assay. Reaction mixtures contained Bud32–Cgi121 (BC) alone (leftmost sample) or BC mixed with increasing concentrations of Pcc1–Kae1 (PK). In the rightmost sample, PK and BC subcomplexes were present in the reaction mixture in equimolar amounts. The reaction mixtures were analyzed by SDS-PAGE, and the radioactivity retained by the proteins was recorded by phosphorimaging (upper panel). The production of free inorganic phosphate for each reaction mixture was monitored by TLC analysis (lower panel).

Bud32 was initially described as an RIO-type serine/threonine protein kinase and its phosphotransferase activity was demonstrated *in vitro* and *in vivo* (30). We therefore tested if the Bud32 ATPase activity corresponded to the protein kinase activity under the conditions used for the synthesis of t^6^A *in vitro*. t^6^A assay was performed using γ-P^32^ ATP, and we measured the radioactivity retained by the different proteins separated on a SDS-PAGE gel (Figure 5) . When the reaction mixture contained only Bud32–Cgi121, a significant autophosphorylation activity of Bud32 was observed, indicating that it can function as a protein kinase under the t^6^A assay conditions. However, the analysis of the complete mixture containing PaSua5 and PaKEOPS showed that, surprisingly, none of the proteins carried a significant level of radioactive signal, indicating a lack of stable protein–phosphate complexes. In line with this observation, the analysis of the same reaction mixture by TLC revealed the release of free inorganic phosphate, indicative of an ATPase activity.

These results indicated that the autophosphorylation activity of Bud32 is modified in the context of the PaKEOPS complex. Because Bud32 interacts with Kae1 in the PaKEOPS complex, we hypothesized that Kae1 might be responsible for the modification of the kinase activity. We thus measured the autophosphorylation activity of Bud32–Cgi121 in presence of increasing concentrations of Pcc1–Kae1 complex. As predicted, the decrease of the phosphorylated Bud32 was proportional to the increase in concentration of Pcc1–Kae1 and was accompanied by an increase in the amount of produced free inorganic phosphate (Figure 5).

In conclusion, our data demonstrated that in the context of PaKEOPS complex, Kae1 switches the activity of Bud32 from kinase into ATPase.

### Nucleotide binding and iron are necessary for the function of Kae1 in t^6^A synthesis

According to structural data (see Discussion), the role of Kae1 during t^6^A synthesis could be to accommodate the reaction intermediate TC-AMP in its active site. We reasoned that TC-AMP will bind in the active site of Kae1 in a similar manner as ATP and that the mutation of active site residues interacting with nucleoside part of ATP should result in total or partial loss of t^6^A activity. To test this hypothesis, we measured the synthesis of t^6^A by the PaKEOPS complex containing Kae1^D159A^ (see previous text). As shown in the Figure 4, the mutant complex lost the capacity to synthesize t^6^A-modified tRNA. As described previously, the reconstituted mutant complex presented wild-type ATPase activity (Figure 3C), indicating that the introduced mutation did not significantly modify the tridimensional structure of Kae1. Moreover, we could partially rescue the t^6^A activity by adding wild-type Kae1 to the mutated complex, suggesting further that the loss of activity was indeed due to Kae1 malfunction (Figure 4). Overall, these data indicate that in the PaKEOPS complex, Kae1 does not function as an ATPase and instead suggest its role in binding of the TC-AMP intermediate.

We next asked if the Fe^3+^ ion in the active site of Kae1/YgjD family of proteins is important for the Kae1 function in t^6^A synthesis. We mutated the strictly conserved H107, which participates in the coordination of Fe^3+^ into alanine. The mutant Pcc1–Kae1^H107A^ binary complex did not present the characteristic absorbance peak at 500 nm, suggesting that Fe^3+^ was not bound in the active site of Kae1. We next measured the t^6^A activity with reconstituted PaKEOPS complex (Kae1^H107A^). The mutant lost ∼90% of the activity as compared with the wild-type complex (Figure 4). As for the Kae1^D159A^ mutant, this activity could be partially rescued by adding wild-type Kae1 to the reaction mixture (Figure 4), and the reconstituted mutant PaKEOPS exhibited normal ATPase activity (Figure 3C). Together, these data indicate that Fe^3+^ is essential for the catalysis of the last step in the t^6^A biosynthesis reaction.

### Molecular docking of TC-AMP into the active site of *P. abyssi* Kae1

To get further support for the role of Kae1 in the binding of TC-AMP, we performed molecular docking of TC-AMP into the active site of Kae1 (see Materials and Methods). Crystal structure of *P. abyssi* Kae1 from which the AMP-PNP ligand was removed was used as template, and the binding of the thermodynamically most favorable conformation of TC-AMP was compared with the observed binding of AMP-PNP. In a control experiment, the docking of PNP-AMP was performed using the same parameters and it showed good agreement with experimental data (Supplementary Figure S4). The computed molar free energy for the best conformation of TC-AMP and AMP-PNP was −9.7 kcal/mol and −9.5 kcal/mol, respectively, suggesting a similar binding affinity for both ligands.

**Figure 6.**
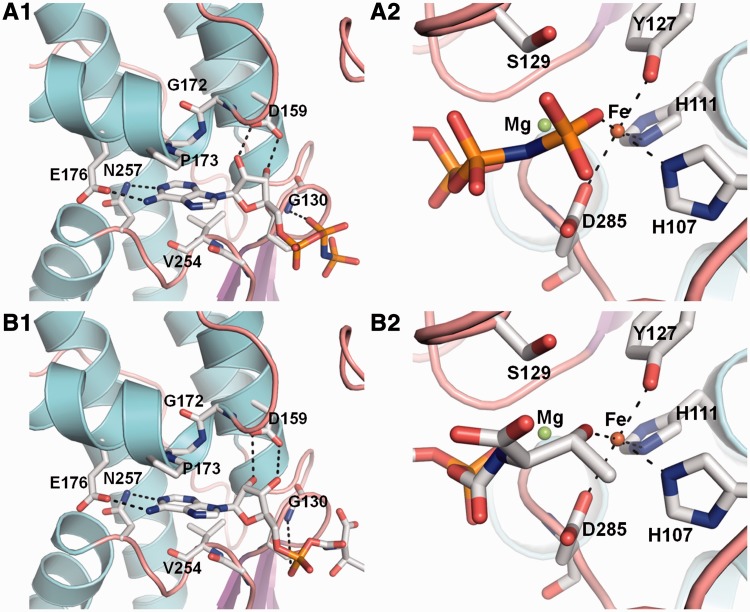
Molecular docking of TC-AMP molecule into the active site of Kae1 from *P. abyssi*. **(A1**) Active site cavity of Kae1 from *P. abyssi* with bound AMP-PNP (PDB file 2IVN). The nucleotide is shown as sticks and hydrogen bonds are indicated by dotted lines. (**A2**) Detailed view of the γ-phosphate binding site. (**B1**) Active site cavity of Kae1 from *P. abyssi* with docked TC-AMP. The thermodynamically most favorable conformation (ΔG = −9.7 kcal/mol) is presented. TC-AMP is shown as sticks and hydrogen bonds are indicated by dotted lines. The docking was performed as blind docking by using Autodock Vina 1.1.2 software (see ‘Materials and Methods’ section). B2. Detailed view of the threonyl binding site. Further details are described in the main text.

The Pa-Kae1 structure in complex with AMP-PNP revealed that the adenine ring makes specific base interactions via its N^6^ and N^1^ atoms to the Glu176 and Asn257 side chains. Both O2’ and O3’ hydroxyl groups from the nucleotide ribose moiety are hydrogen-bonded to the Asp159 carboxylate. In addition, the 2’OH group of the ribose moiety interacts with the Gly172 amide group. In the predicted binding of TC-AMP, the nucleoside part of the intermediate could be well superposed with AMP-PNP, such that all of the mentioned interactions were preserved (Figure 6, Supplementary Figure S5). Differences were observed for the binding of the l-threonyl-carbamoyl part of the molecule. The side-chain -OH group of the threonyl moiety interacted with His107 N2, Tyr127 OH, Ser129 OH and the Asp285 carboxylate group. The carboxylate group of the threonyl moiety was contacted by the main chain amide from Gly131, and the α-phosphate (AMP part of the molecule) by the main amide from both Asp285 and Gly253 (and also probably by a Mg^2+^ ion identified in the Pa-Kae1 structure from residual electronic density next to the iron atom) (Supplementary Figure S5). Interestingly, the side-chain -OH group of threonyl moiety is predicted to participate in the coordination sphere of Fe^3+^, indicating that iron atom is directly involved in the binding of TC-AMP. Taken together, these data are consistent with the role of Kae1 in binding of TC-AMP intermediate in course of the biosynthesis of t^6^A modification.

### Pcc1 and Kae1 are mainly responsible for the binding of tRNA

**Figure 7.**
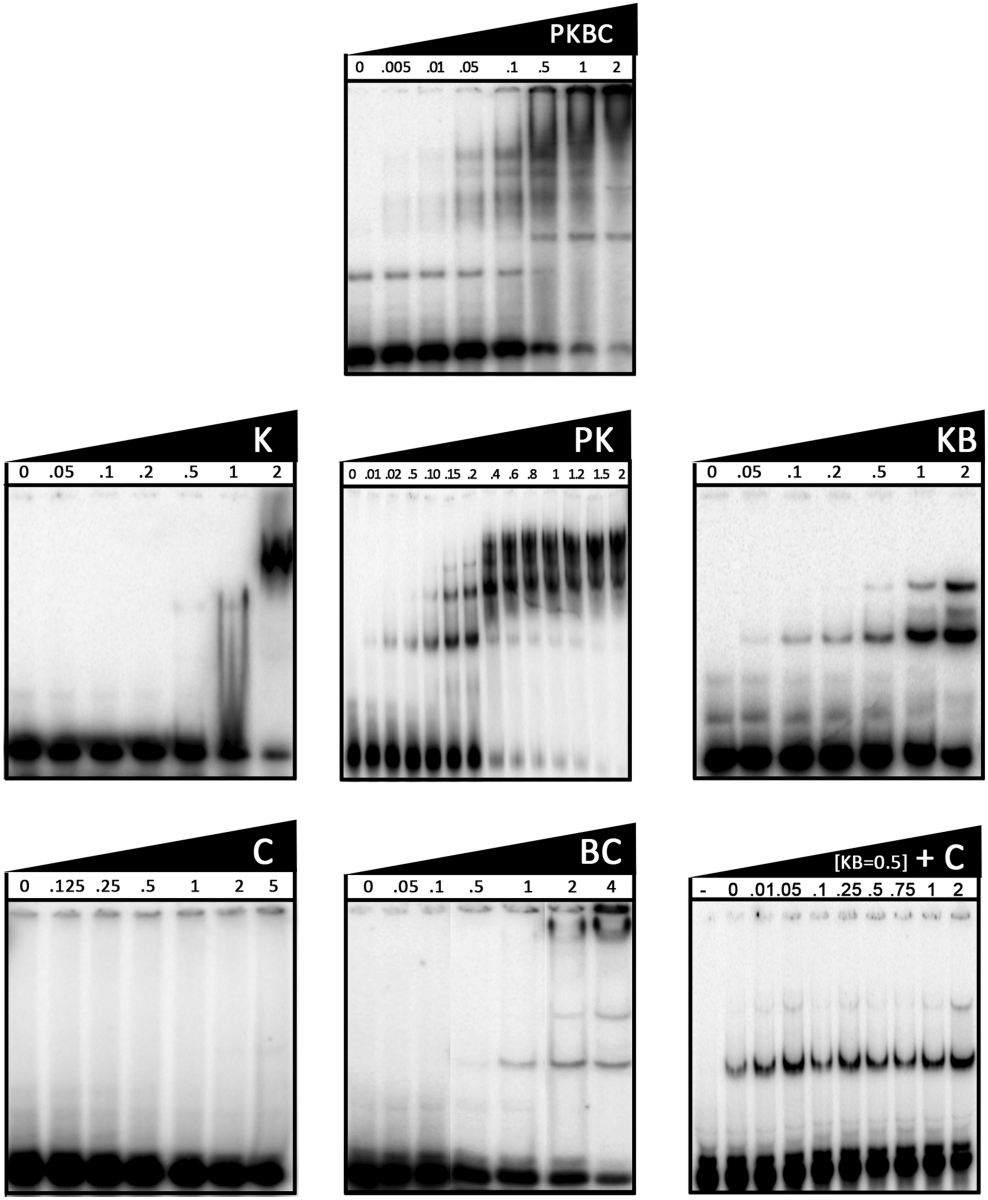
Binding of tRNA by PaKEOPS subcomplexes and individual proteins. Individual PaKEOPS subunits and subcomplexes were mixed with 10 nM of radiolabeled (P^32^) tRNA at room temperature and the mixture was separated by native PAGE (see ‘Materials and Methods’ section). The radioactive bands corresponding to unbound tRNA (at the bottom of each gel) and the nucleoprotein complexes were recorded by phosphorimaging. Protein concentrations used are indicated in µM at the top of each panel. In the downright panel, fixed concentration of the Kae1–Bud32 complex (0.5 µM) was titrated with increasing concentrations of Cgi121, which are indicated for each sample at the top of the gel. Minus sign stands for the control sample to which proteins were not added. Single letter designations are as follows: P, Pcc1; K, Kae1; B, Bud32 and C, Cgi121.

We previously demonstrated that the PaKEOPS complex binds tightly to tRNA; however, the contribution of each subunit in the binding process was unknown (23). We used electrophoretic mobility shift analysis (EMSA) experiments to directly measure binding of different PaKEOPS subunits to radioactively labeled substrate tRNA and compare their binding profile relative to the whole PaKEOPS complex (Figure 7). Formation of a discrete shifted band was visible already at 10 nM of PaKEOPS (corresponding to molar ratio protein:tRNA of 1:1) with apparent Kd value of 100–500 nM (corresponding to 50% of bound tRNA). Using Kae1 alone, we observed formation of diffuse bands of retarded tRNA starting from 1 µM concentration of protein, and almost the entire probe was retarded at 2 µM of Kae1.

Different from Kae1, using the binary complex Pcc1–Kae1, we could observe progressive formation of several retarded bands, corresponding to different forms of nucleoprotein complexes. The formation of complexes started already from 10 nM of protein concentration and apparent Kd value was in the range between 200 and 400 nM. Thus, the tRNA binding profile of Pcc1–Kae1 is very similar to the one observed for the whole PaKEOPS complex.

Progressive appearance of retarded bands was also observed for the Kae1–Bud32 complex, starting from 50 nM of protein concentration. The apparent Kd value for tRNA of this complex was approximately at 2 µM of protein concentration, indicating that this complex has less affinity for tRNA compared with Pcc1–Kae1 or the whole PaKEOPS complex.

The third binary complex Bud32–Cgi121 exhibited less pronounced retardation of tRNA; the formation of nucleoprotein complexes was observed at 1 µM of protein. At higher protein concentration, most of the radioactivity was observed in the well of the gel, indicating non-specific binding and the formation of aggregates.

Finally, using Cgi121 alone, we could not detect the formation of nucleoprotein complexes even at very high protein to tRNA ratios. This result indicated that Cgi121 does not participate directly in tRNA binding. As Cgi121 has a stimulating effect for the formation of t^6^A (see earlier text), we asked if Cgi121 acted by increasing the affinity of Bud32 and/or other PaKEOPS proteins for tRNA. To test this hypothesis, we performed an EMSA experiment by incubating fixed concentration of Kae1–Bud32 with increasing concentrations of Cgi121 (Figure 7). The addition of Cgi121 did not change the migration profile or the quantity of the retarded probe at any of the tested concentrations.

## DISCUSSION

In this work, we dissected the PaKEOPS complex into its individual components and studied their function and contribution to the total reaction catalyzed by the complex in course of the biosynthesis of t^6^A. Using this approach, we could determine a minimal set of proteins required for the reaction and assign functions for each of the individual proteins.

### Pcc1, Kae1 and Bud32 are required and sufficient for the *in vitro* biosynthesis of t^6^A

The requirement of different subunits of KEOPS for t^6^A formation was initially studied *in vivo* in *S. cerevisiae* by gene deletion (24,25). Severe growth phenotypes were reported for *pcc1*, *bud32* and *kae1* deletions, whereas the deletion of *cgi121* resulted in a mild phenotype. These data are consistent with our *in vitro* results, which show that Pcc1, Kae1 and Bud32 are absolutely required for the synthesis of t^6^A, whereas Cgi121 is dispensable. However, a recent genetic study in halophilic archaeon *Haloferax volcanii* reported only a mild t^6^A phenotype (the mutant strain retained 80 % of t^6^A levels as compared with the wild type) for the pcc1 deletion mutant, whereas Cgi121 was shown to be essential (31). This suggested that the latter protein might be involved in other cellular function(s) in *H. volcanii* cells. The explanation for the discrepancy between this study and our *in vitro* results is presently unclear; although, it is possible that KEOPS functions differently because Kae1 and Bud32 are fused in a single polypeptide in *H. volcanii* and/or because the complex operates at the high salt concentrations present in *Haloferax* cells.

### Cgi121 is an accessory chaperone protein that regulates the t^6^A catalytic activity

Although not strictly necessary for the formation of t^6^A, the addition of Cgi121 to the mixture of Pcc1, Kae1 and Bud32 had a stimulating effect on the synthesis of this modification. This is probably not due to an increased tRNA binding capacity, as the addition of Cgi121 to the Kae1–Bud32 complex did not improve the affinity for tRNA. Rather, Cgi121 seems to be required for the assembly of KEOPS complex via formation of stable interactions between the subunits. This notion is based on an observation that we failed to isolate a stable Pcc1–Kae1–Bud32 complex by mixing Pcc1–Kae1 and Kae1–Bud32 binary complexes, although this mixture catalyzed the formation of t^6^A ,indicating the formation of a transient Pcc1–Kae1–Bud32 complex. Consistent with our data, the structural study of KEOPS complex reported only the purification of the Kae1–Bud32–Cgi121 ternary complex (2). The structure of this complex revealed that interaction of Cgi121 with Bud32 altered significantly the conformation of Bud32, suggesting that binding of Cgi121 to Bud32 might provoke a chain reaction of conformational changes necessary for the formation of the whole KEOPS complex. Taken together, these observations indicate that Cgi121 does not participate directly in the catalytic activities of KEOPS; instead, it regulates these activities by acting as an allosteric effector. Consistent with this notion, Cgi121 has a short half life in the cells of *S. cerevisiae* (∼30 min) and its average abundance is 10-fold lower as compared with Kae1 and Bud32 (PaxDb database) (2,37).

In bacteria, the functional counterparts of the KEOPS complex are proteins YgjD, YeaZ and YjeE (DEZ complex), which together catalyze the transfer of threonyl-carbamoyl to tRNA (16,17,23). However, unlike KEOPS, the three proteins seem not to form readily a stable complex when mixed together (16). Interestingly, a recent study reported for the first time a calorimetric evidence for the formation of a ternary DEZ complex that was dependent on the presence of ADP (22). It appears therefore that two different strategies were developed to stabilize the complexes that catalyze the last step of the t^6^A synthesis, i. e. the bacterial system uses the binding of ADP, whereas the archaeal and eukaryal systems use an additional protein, Cgi121.

### Pcc1 functions as a dimerization module

Pcc1 was previously shown to form homodimers in solution, and purified Kae1–Pcc1 complex revealed a 2:2 binding stoichiometry, suggesting that Pcc1 functions as a dimerization module in the presence of other KEOPS subunits (2). In support of this model, we have previously reported that the apparent molecular mass of PaKEOPS could correspond to a dimer (23). This notion is now further reinforced by the observation that the Kae1–Bud32–Cgi121 ternary complex is monomeric in solution, indicating that Pcc1 is required for dimerization.

Interestingly, YgjD and its paralog YeaZ form heterodimers in solution, and similarly, Qri7 was recently shown to form dimers in solution (20,22,34). In contrast to these proteins, Kae1 is monomeric in solution. It is therefore tempting to speculate that Pcc1 was recruited in the course of evolution to maintain the dimerization capacity of Kae1. Alternatively, the homolog of Pcc1 was lost in bacteria after the duplication of the ancestral gene coding for YgjD.

*In vivo* study in *S. cerevisiae* demonstrated that point mutations at the Pcc1 dimer interface resulted in severe growth phenotype, comparable with that observed with the Pcc1 deletion mutant (2). These data indicated that KEOPS must function as a dimer, but the question of why it must do so remains open. One possibility might be that the efficient binding of tRNA substrate by KEOPS requires dimerization. Indeed, we have shown that binding of tRNA to KEOPS occurs in a cooperative manner, suggesting that binding of one molecule of tRNA to the KEOPS dimer facilitates the binding of the second one (30). Similar model was proposed for the seryl-tRNA synthetase, a homodimeric enzyme for which cooperative cross-domain tRNA binding was demonstrated using biochemical and structural approaches (38–40).

### Kae1 is a threonyl-carbamoyl transferase with an iron-dependent mode of substrate binding and recognition

Crystal structures of HypF and TobZ proteins showed that carbamoyl-adenylate catalytic intermediates produced by YrdC-like domain of these proteins were channeled across the interface with Kae1-like domain and bound in its active site (41,42,43). Tobramycin was also found positioned in the active site cavity of the Kae1-like domain next to the nucleotide binding site, indicating that this domain is responsible for the transfer of the carbamoyl group to the final acceptor molecule—tobramycin (41). Comparison of *P. abyssi* Kae1 structure liganded to AMP-PNP with HypF and TobZ structures showed that the nucleotide binding residues in the active sites of these proteins are conserved. Based on these data, we proposed an analogous model for the function of Kae1 in t^6^A synthesis, in which Kae1 binds TC-AMP in its active site and transfers the l-threonyl-carbamoyl moiety to tRNA (23). Consistent with this model, we showed in the present study that mutating conserved residue involved in nucleotide binding of *P. abyssi* Kae1 totally abolished the t^6^A activity. In line with this result, the equivalent mutation in mitochondrial Qri7 protein resulted in total loss of t^6^A activity (34). Point mutations targeting nucleotide binding site of Kae1 of *S. cerevisiae* exhibited severe growth phenotype comparable with the null mutant, indicating good correlation between *in vivo* and *in vitro* data. As the nucleotide binding residues are conserved in the Kae1/YgjD/ Qri7 family, it is likely that our data can be extrapolated to all members of this family of proteins. Our data further suggest that the low ATPase activity of Kae1, which can be measured *in vitro* (2,19), is not physiologically relevant.

All members of the Kae1/YgjD/Qri7 family possess two histidine residues that participate in the coordination sphere of iron (Kae1) or zinc (YgjD) atom in the active site (19). In the structure of *P. abyssi* Kae1, the iron atom is directly linked to AMP-PNP via the gamma phosphate, which is a unique feature among the members of the ASHKA family. It was proposed that such a singular way of nucleotide binding would be important for the function of Kae1, but its exact role remained mysterious. In this study, we mutated one of the conserved histidine residues, which resulted in a loss of ∼90% of t^6^A activity, indicating that iron has an essential role in t^6^A synthesis. Consistent with these data, cells of *S. cerevisiae* were reported to be non-viable when residues that are involved in binding of iron are mutated (His110, His106, Asp284, *S. cerevisiae* numbering) (2,25). In addition, mutation of metal-binding residues of Qri7 severely impaired t^6^A formation, indicating that the function of metal ion in t^6^A synthesis is a common feature of this family of proteins (34).

Assuming that the nucleotide backbone of TC-AMP binds in the Kae1/YgjD/Qri7 active site in the same way as the ATP analog, the iron/zinc atom would be well placed to interact directly with the carbamoyl-threonine moiety (19,34,41). We have tested this hypothesis by docking the TC-AMP molecule into the active site of PaKae1. The thermodynamically most favorable conformation of TC-AMP (ΔG = −9.7 kcal/mol) could be well superposed with AMP-PNP bound in the active site (Figure 6, Supplementary Figure S4). In this conformation, hydroxyl group of the threonyl moiety established direct interaction with the iron.

Interestingly, threonyl-tRNA synthetase (ThrRS) binds ATP and threonine to form a threonyl-adenylate intermediate and carries zinc ion in its active site (44,45). The zinc ion is directly involved in threonine recognition by forming a pentacoordinate intermediate with the amino group and side-chain hydroxyl group of threonine, thus preventing misactivation of isosteric valine (44). In analogy to ThrRS, we propose that the metal ion in the active site of the Kae1/YgjD/Qri7 family of proteins is neither strictly catalytic nor structural, but instead is directly involved in TC-AMP recognition.

Whereas the Kae1/YgjD/Qri7 family possesses invariant HxxxH motif implicated in metal binding, in ThrRS, zinc is liganded by two histidine residues that are far away in the primary sequence (His385 and His511, *E. coli* ThrRS numbering). This suggests that the metal-based mechanism for the recognition of threonine was acquired independently in two unrelated protein lineages and thus illustrates another case of convergent evolution.

### Kae1 and Pcc1 form the tRNA binding core of the KEOPS complex

Using EMSA experiments, we showed that the tRNA binding profile of the Pcc1–Kae1 complex was comparable with that of the whole KEOPS complex. Moreover, the Bud32–Cgi121 complex showed low affinity for tRNA, whereas Cgi121 did not bind at all to tRNA. Taken together, these data indicate that the Pcc1–Kae1 complex is mainly responsible for tRNA binding, whereas Bud32 possibly plays a minor role. The predicted function of Kae1 in transfer of threonyl-carbamoyl moiety implies that the anticodon loop of tRNA carrying the A37 nucleotide binds in the active site of Kae1. The finding that Kae1 binds tRNA directly fits well with this proposal. *In vivo* substrate specificity study of t^6^A formation using *Xenopus laevis* oocytes demonstrated that in addition to the targeted A37, only U36 and A38 were required for quantitative conversion of A37 to t^6^A37 (46). These nucleotides are in immediate proximity to A37 and should presumably dock in the active site of Kae1, suggesting that this protein is the principal element responsible for substrate recognition.

Because Pcc1 was insoluble when expressed in absence of Kae1, it was not possible to conclude whether Pcc1 directly binds tRNA or it acts indirectly by increasing the affinity of Kae1 for tRNA. However, structural data support the direct role of Pcc1 in tRNA binding. Indeed, structure of Pcc1 revealed a KH-like domain that functions in binding of RNA, ssDNA and hairpin RNA in a variety of proteins (2). Typically, the nucleic acid binding capacity of this domain relies on the presence of conserved loop motif GxxG, which is absent in Pcc1. However, diverged functional members of this family lacking this motif have been discovered (48), thus maintaining the possibility that Pcc1 could interact directly with tRNA. Interestingly, only four unpaired nucleotides can be accommodated by KH domains, indicating that Pcc1 might bind to loop regions of tRNA molecules (48).

### Bud32 functions as a P-loop ATPase and its activity is essential for the biosynthesis of t^6^A

The presence of Bud32 in the KEOPS complex was puzzling, as it was difficult to accommodate kinase activity in the reaction scheme leading to the synthesis of t^6^A modification. In this study, we demonstrated that in the context of the KEOPS complex, Bud32 does not function as a bona fide kinase but as an ATPase. We further confirmed previous observations that Kae1 modulates the kinase activity of Bud32, thus switching Bud32 from ‘kinase mode’ to ‘ATPase mode’ (27). These results are in contradiction with the data reported by Mao and colleagues, who showed that Kae1 was a substrate for Bud32 (2). The reasons for this discrepancy are unclear for the moment.

Mutational analysis of the catalytic residue of Bud32 established that Bud32 is responsible for the observed ATPase activity of KEOPS (hydrolysis of ATP to ADP and inorganic phosphate) and that this activity is essential for the synthesis of t^6^A. Although the bacterial DEZ complex lacks a Bud32 homolog, it also hydrolyzes ATP to ADP and phosphate, but, unlike KEOPS, this activity is not stimulated by tRNA (16). Interestingly, it was recently demonstrated that *Bacillus subtilis* DEZ proteins catalyze formation of t^6^A from TC-AMP in the absence of ATP, indicating that ATPase activity of DEZ is not required for t^6^A synthesis in bacteria (17). Thus, these data revealed the existence of mechanistic differences between the bacterial and archaeal/eukaryotic systems concerning the final step of the reaction leading to t^6^A-modified tRNA.

What is the function of the ATPase activity of Bud32? We hypothesize that this activity is required for the dissociation of tRNA from the complex once the addition of threonyl-carbamoyl is completed. This hypothesis is inspired by a recent report on another atypical RIO-type kinase (Rio2) that, like Bud32, lacks the structural elements required for substrate recognition. Similar to Bud32, Rio2 does not function as a kinase but it exhibits robust ATPase activity *in vitro* (49). Interestingly, the energy released by ATP hydrolysis is required for dissociation of the Rio2 from the ribosome, a necessary step in pre-40S particle maturation (49). Moreover, Rio2 contains an unusual phosphoaspartate intermediate typically found in P-type ATPases but not in protein kinases. Phosphoryl transfer from ATP to Asp257 in Rio2’s active site and subsequent hydrolysis of the aspartylphosphate was proposed to be a trigger to power 40S subunit biogenesis (49). Remarkably, Asp257 is conserved in the Bud32 active site (corresponding residue in *P. abyssi* Bud32 is Asp144), suggesting that Bud32 could also function in the same manner as P-type ATPases.

RIO family kinases are ubiquitously distributed in the three domains of life, emphasizing their essential function. However, the physiological role of prokaryotic RIO family kinases remains obscure (50). To the best of our knowledge, Bud32 and Rio2 are the first two examples of atypical RIO-type kinases for which the lack of kinase activity was demonstrated in the context of their respective biosynthetic processes. This raises the intriguing possibility that the atypical RIO-type kinases function as ATPases rather than *bona fide* kinases. Nevertheless, it should be kept in mind that Bud32 may function as a protein kinase *in vivo* if it is not bound to Kae1. On the other hand, in the organisms in which Kae1 and Bud32 are expressed as a single fusion polypeptide (e. g. *Methanococcus jannashii and Haloferax* species), the Bud32 kinase activity is most likely inhibited.

The proposed function of Bud32 ATPase activity in facilitating dissociation of tRNA provides an explanation for the essential character of this activity. Indeed, the *P. abyssi* KEOPS complex co-purifies with tRNA of *E.coli*, suggesting formation of a stable nucleoprotein complex in the natural host (23). Thus, the dissociation of KEOPS complex from tRNA could require energy released by ATP hydrolysis. Similar dissociation mechanism is exploited by the elongation factor Tu (dubbed EF1A in Archaea and Eukarya), which is a GTPase that positions aminoacyl-tRNA complexes within the A-site of prokaryotic ribosomes. Once the aminoacyl-tRNA complex is positioned in the ribosome via codon–anticodon base-pairing, EF-Tu undergoes a conformational change powered by GTP hydrolysis that results in the dissociation of aminoacyl-tRNA from EF-Tu (51)*.* The bacterial DEZ complex, on the other hand, does not co-purify with tRNA and it shows low binding affinity for tRNA *in vitro*, indicating formation of unstable nucleoprotein complexes (not shown, unpublished data). Consequently, energy would not be required for the dissociation of tRNA, which could explain why DEZ complex functions without addition of ATP in the reaction mixture (17).

The C-terminal tail of Bud32 could be involved in binding and dissociation of tRNA during t^6^A synthesis. Model structure of the entire KEOPS complex showed that the C-terminal tail of Bud32 binds in the catalytic cleft of Kae1 directly adjacent to the metal ion coordinating center. This leaves the tail well-placed to influence the binding of tRNA, which should occur at the entrance of the catalytic cleft next to the metal binding site. The observed tRNA binding profile of the Bud32–Cgi121 complex indicated that Bud32 is marginally involved in the binding of tRNA, which fits the idea that only C-terminal tail might be directly involved in binding. Interestingly, Mao and colleagues reported that the deletion of the C-terminal tail did not prevent the binding of Bud32 to Kae1, but the cells of *S. cerevisiae* carrying the same mutation exhibited a severe growth phenotype (2). Taken together, these observations are compatible with a possible role of the Bud32 tail in regulating tRNA binding to the KEOPS complex.

### Putative mechanistic model for the catalysis of the threonyl-carbamoyl transfer to tRNA by KEOPS

**Figure 8.**
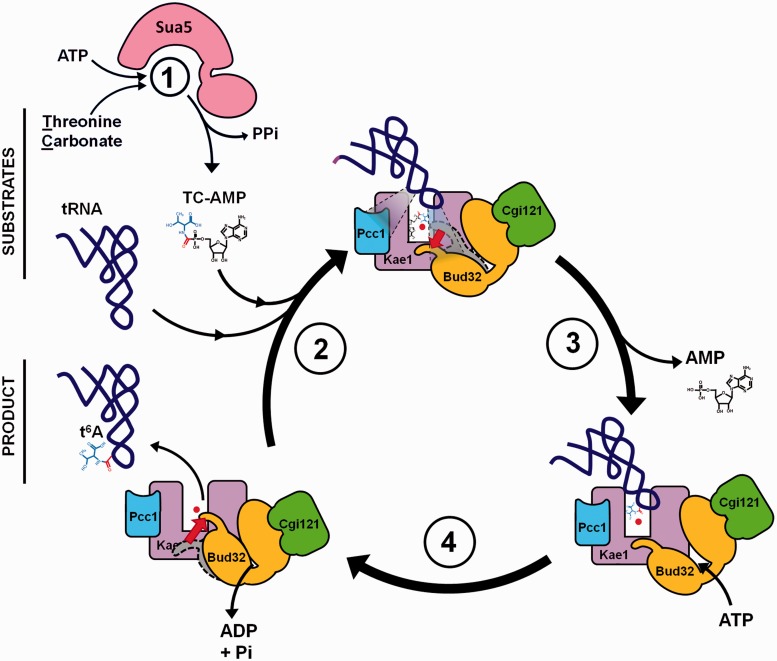
Putative mechanism for the catalysis of the last step in the biosynthesis of t^6^A modification by the KEOPS complex. (1) *Formation of TC-AMP*: Sua5 catalyzes the condensation of threonine, bicarbonate and ATP leading to the formation of an unstable TC-AMP intermediate and release of inorganic pyrophosphate. (2) *Binding of tRNA and TC-AMP to KEOPS*: TC-AMP binds into the active site of Kae1 and interacts directly with the iron atom via threonyl part of the molecule. Binding of tRNA to the complex provokes conformational changes in the complex, including the movement of the C-terminal tail of Bud32 (indicated with an arrow). Pcc1 and Kae1 are involved in the major part of contacts (gray triangles) with tRNA, whereas Bud32 participates in binding of tRNA via C-terminal tail. Anticodon loop carrying the target nucleotide A37 is positioned at the entrance to the active site cavity of Kae1 next to the TC-AMP intermediate and iron atom. (3) *Transfer of* l-*threonyl-carbamoyl to tRNA*: Threonyl-carbamoyl moiety is transferred to A37 of substrate tRNA in an ATP-independent fashion and AMP is released. (4) *Release of t^6^A-modified tRNA*: ATP hydrolysis catalyzed by Bud32 powers the conformational changes, in particular motion of the C-terminal tail of Bud32 (indicated by red arrow), which leads to the dissociation of modified tRNA from the KEOPS complex. The resulting KEOPS complex is competent for another catalytic cycle. Further details are given in the main text.

Based on the combination of biochemical data presented in this work and previous structural studies of the KEOPS complex, we propose a putative *modus operandi* for the catalysis of the last step in the biosynthesis of t^6^A modification (Figure 8).

The TC-AMP intermediate produced by Sua5 is delivered into the active site of Kae1 probably during a transient interaction between Sua5 and Kae1. The metal ion bound in the active site of Kae1 participates in binding and recognition of the TC-AMP intermediate by forming coordination bonds with the side-chain hydroxyl group of threonine. tRNA molecule binds to KEOPS in such a way that the major identity determinants U36 and A38 as well as the target nucleotide A37 are bound in the catalytic cleft of Kae1 next to the TC-AMP. Pcc1 enhances tRNA binding either by binding directly to tRNA or through dimerization process, whereas the C-terminal tail of Bud32 might participate in the binding of the anticodon stem. The cognate tRNA molecule is recognized by the specific interactions between the residues in the Kae1 active site and the relevant anticodon loop bases. Kae1 catalyzes the transfer of the l-threonyl-carbamoyl moiety to the adenosine base of A37 without ATP hydrolysis, thus the role of Kae1 in this reaction is to bring the reactants together in a favorable orientation for the reaction to occur. Binding of tRNA to Kae1 provokes conformational changes that are propagated to Bud32 and stimulate its ATPase activity. The energy liberated by ATP hydrolysis is used to power conformational changes in Bud32, leading to the release of tRNA perhaps via the motion of the C-terminal tail of Bud32. This model requires that in the case of cognate tRNA, the ATP hydrolysis is sufficiently delayed to allow for the transfer of TC-AMP to occur before the dissociation of tRNA from the complex.

### Concluding remarks

In conclusion, we show here that KEOPS is a sophisticated molecular machine in which the core catalytic protein Kae1 is associated to the three accessory proteins Pcc1, Bud32 and Cgi121, each of which contributes distinct and important functions in dimerization, tRNA binding, structuration of the complex and regulation of its activity. Overall, our data provide significant advances in the comprehension of the functioning of this complex in the synthesis of the universal tRNA modification and a rational framework for future structural studies and in-depth mechanistic studies.

Recently, a minimal system catalyzing the formation of t^6^A-modified tRNA in mitochondria of *S. cerevisiae* was reconstituted *in vitro*. This system consists of Sua5 and Qri7 (mitochondrial homolog of Kae1), indicating that Qri7 can alone substitute for KEOPS (34). This discovery strengthens further the proposed evolutionary scenario in which Sua5 and Kae1 were the original system present in the last universal common ancestor (23). In this scenario, the original two-component system evolved toward greater complexity in bacterial and archaeal/eukaryal branches by acquiring specialized accessory proteins. These proteins were perhaps necessary for the fine tuning of the catalytic process, rendering it more accurate and/or more efficient to cope with the increasing complexity of cellular life forms. Future comparative studies of the minimal mitochondrial system and the two modern complex systems will shed light onto the adaptive forces driving the evolution of these remarkable molecular machines.

## SUPPLEMENTARY DATA

Supplementary Data are available at NAR Online, including [52].

## FUNDING

Funding for open access charge: This work was supported by the Agence Nationale de Recherche [ANR-09-BLAN-0349 to P.F.]; a Ph.D. fellowship from ENS Lyon (to L.P.).

*Conflict of interest statement*. None declared.

## Supplementary Material

Supplementary Data
