# Supplementary material for: Functional assignment of KEOPS/EKC complex subunits in the biosynthesis of the universal t6A tRNA modification
Source: Nucleic Acids Res. 2013 Aug 14;41(20):9484–99. doi: 10.1093/nar/gkt720 (PMC3814370; doi:10.1093/nar/gkt720)
Supplement: Supplementary Data [file supp_41_20_9484__index.html]

Functional assignment of KEOPS/EKC complex subunits in the biosynthesis of the universal t6A tRNA modification — Functional assignment of KEOPS/EKC complex subunits in the biosynthesis of the universal t6A tRNA modification — Supplementary Data 

# Functional assignment of KEOPS/EKC complex subunits in the biosynthesis of the universal t6A tRNA modification

## Supplementary Data

files

**Files in this Data Supplement:**

- Supplementary Data - pdf file
