# Supplementary material for: Functional assignment of KEOPS/EKC complex subunits in the biosynthesis of the universal t6A tRNA modification
Source: Nucleic Acids Res. 2013 Aug 14;41(20):9484–99. doi: 10.1093/nar/gkt720 (PMC3814370; doi:10.1093/nar/gkt720)
Supplement: Supplementary Data [file supp_gkt720_nar-01567-r-2013-File010.pdf]

SUPPLEMENTARY DATA

Supplementary methods

Cloning and expression of recombinant ternary complex Kae1-Bud32-Pcc1

Four different strategies were employed to clone and express the recombinant ternary complex Kae1-Bud32-Pcc1:

(1) KEOPS expression plasmid pET28::KEOPS(His) (Perrochia et al. 2013) was digested with *Sac*I and *Sal*I restriction enzymes to remove the gene encoding Cgi121. The resulted linear DNA was converted into blunt end DNA using Klenow fragment (NEB) and then self-ligated.

(2) DNA fragment encoding Kae1 and Bud32 was amplified by PCR using pET28::KEOPS(His) as template. The amplified fragment was gel purified (NucleoSpin, Macherey and Nagel) and cloned into pET26::Pcc1 expression plasmid *via Nde*I and *Xho*I restriction sites.

(3) Kae1-Pcc1 binary complex and Bud32 were co-expressed in *E. coli* Rosetta 2 strain using compatible plasmids pET26b::Kae1-Pcc1 (with hexa histidine tag on Pcc1) and pCDFDuet::Bud32, respectively.

(4) The polycistronic sequence encoding Kae1, Bud32 and Pcc1 was synthesized chemically (Genscript) and cloned into pET28a expression vector via *Nde*I and *Xho*I restriction sites. Each coding sequence was preceded by an *Escherichia coli* ribosome binding site (5'-TTAACTTTAAGAAGGAG). The *pcc1* gene was fused to a sequence encoding hexa-histidine tag at its 3' end.

Table S1. Cloning of genes used in this study.

**Genes$^{\#}$**	**Cloning strategy**	**Template DNA**	**Expression vector**	**Restriction site for cloning**	**Restriction sites for digestion**
***Pa*Bud32^{-H}**	PCR	*P. abyssi genomic DNA*	pET9aSn1	*Nde*I/*Not*I	
***Pa*Cgi121^{-H}**	Gene synthesis		pET26b	*Nde*I/*Not*I	
***Pa*Pcc1^{-H}**	Digestion	pET28a::KEOPS	pET26b	*Nde*I/*Xho*I	*Nde*I/*Xho*I
**P^{-H}K**	Digestion	pET28a ::KEOPS	pET28a		*Xba*I/*Sal*I*****
**KB^{-H}**	PCR	pET28a ::KEOPS	pET26b	*Nde*I/*Xho*I	
**BC^{-H}**	PCR	pET28a ::KEOPS	pET26b	*Nde*I/*Xho*I	

**KBC^{-H}**	PCR	pET28a ::KEOPS	pET26b	*Nde*I/*Xho*I	

# Single letter designations correspond to: P, *pcc1*, K, *kae1*, B, *bud32* and C, *cgi121*.

* after digestion the two ends of the linearised vector were blunted using Klenow fragment (NEB) and ligated.

Table S2. Oligonucleotide primers used for site-directed mutagenesis.

Primer	Sequence (5'->3')	Nucleotide substitution
Kae1D159A-F	AATCGGAAACGCCATAG**C**TGTCTTTGCAAGGGAAC	a476c
Kae1D159A-R	GTTCCCTTGCAAAGACA**G**CTATGGCGTTTCCGATT	a476c
Kae1H107A-F	ACCGATAGTTGGGGTAAAC**GC**CTGTATAGCCCATGTAGAG	c319g - a320c
Kae1H107A-R	CTCTACATGGGCTATACAG**GC**GTTTACCCCAACTATCGGT	c319g - a320c
Bud32D127R-F	GCGGGGATAGTTCACGGG**CG**CTTAACGACCTCAAATAT	g379c - a380g
Bud32D127R-R	ATATTTGAGGTCGTTAAG**CG**CCCGTGAACTATCCCCGC	g379c - a380g

Buffer composition

Lysis Buffer (LBf): Tris-HCl 20 mM, pH 8.0, NaCl 200 mM, β-mercaptoethanol 5 mM, imidazole 10mM

Storage Buffer (LBs): Tris-HCl 20 mM, pH 8.0, NaCl 200 mM, glycerol 10% (v/v)

Elution buffer (EB): 0,5 M ammonium acetate, 10mM magnesium acetate, 0,1% SDS, 1mM EDTA,

Reaction Buffer (RB): 50 mM Tris-HCl pH8, 35 mM KCl, 5 mM DTT, 1 mM ATP, 10 mM NaHCO3-

Binding Buffer (BB) : 20 mM Tris-HCl pH 7.5, 100 mM NaCl, 1mM DTT

TGE Buffer: 50 mM Tris-HCl pH 7.5, 8 mM glycine, 0.1 mM EDTA

TBE buffer: 89 mM Tris-HCl pH8, 89 mM Boric acid, 2 mM EDTA

Supplementary figures

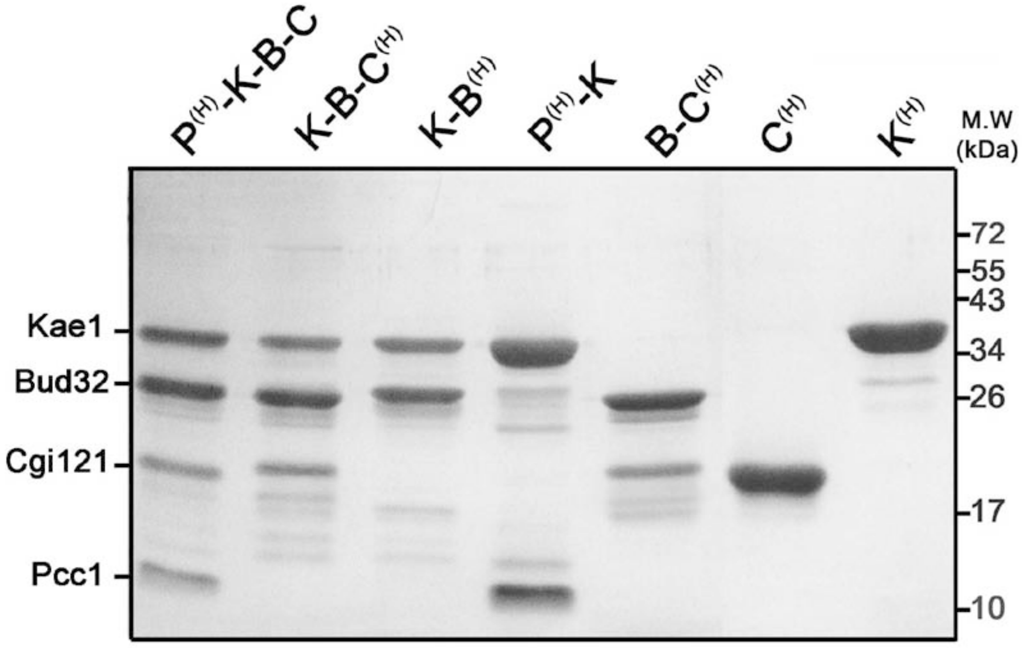

Figure S1. SDS-PAGE analysis of subcomplexes of KEOPS from *P. abyssi*

The recombinant subcomplexes were purified from overexpressing *E. coli* Rosetta cells by Ni-NTA affinity chromatography followed by gel filtration. An aliquot of an elution fraction corresponding to a single peak containing the subcomplexes was loaded onto SDS-PAGE for analysis. The single letter designations are as follows: P - Pcc1, K - Kae1, B – Bud32, C – Cgi121, H – presence of hexahistidine tag.

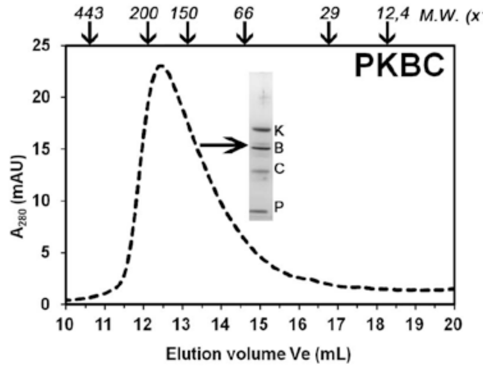

	theorical M.W. (kDa)
**P**cc1	9,8
**K**ae1	35,4
**B**ud32	26,3
**C**gi121	19

	theorical M.W. (kDa)	elution volume (mL)	apparent M.W. (kDa)
**PK**	45,2	14,7	66
**BC**	45,3	16,1	35
**KB**	61,7	16,5	30
**KBC**	80,7	14,6	69
**PKBC**	90,5	12,5	190

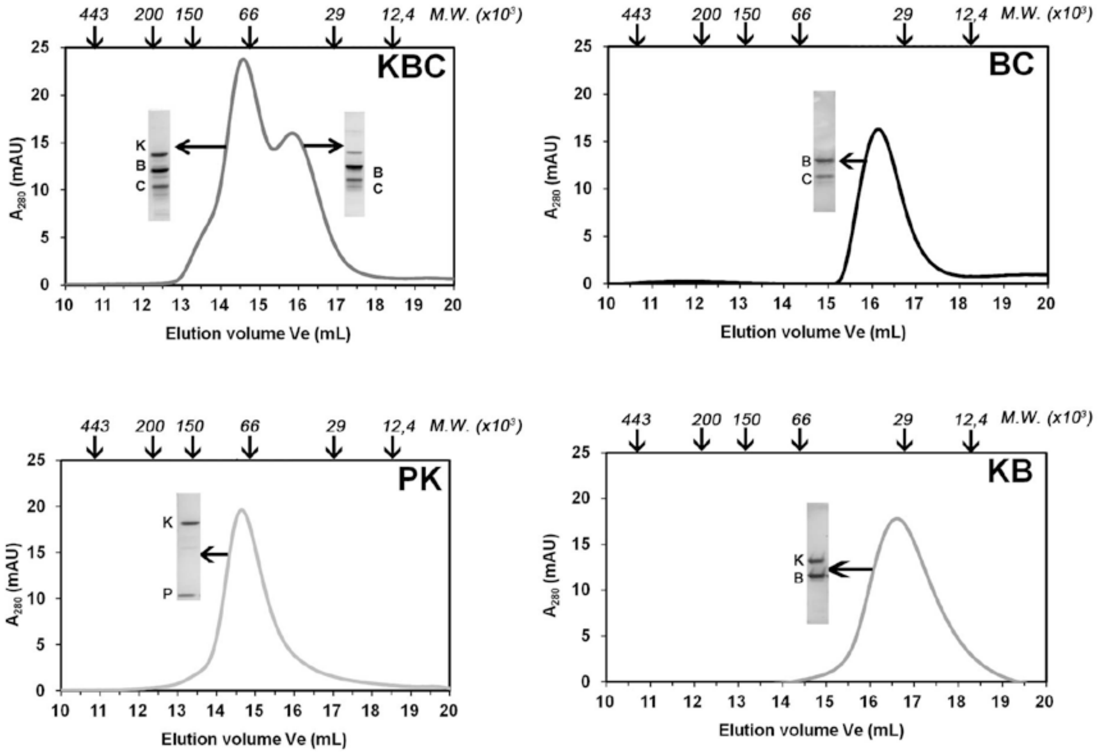

Figure S2. Elution profile of KEOPS subcomplexes after purification by gel filtration.

The recombinant subcomplexes were partially purified from overexpressing *E. coli* Rosetta cells by Ni-NTA affinity chromatography. The fractions containing subcomplexes were pooled and loaded on Superdex 200 HR10/300 prepacked column. SDS-PAGE analysis is shown next to each peak. In the table in the top right panel are indicated the calculated theoretical molecular masses for different subcomplexes, the observed elution volumes and corresponding apparent molecular masses. The molecular masses of the reference proteins used for the calibration of the column are indicated in kDa at the top of each elution profile. The single letter designations are as follows: P - Pcc1, K - Kae1, B – Bud32, C – Cgi121.

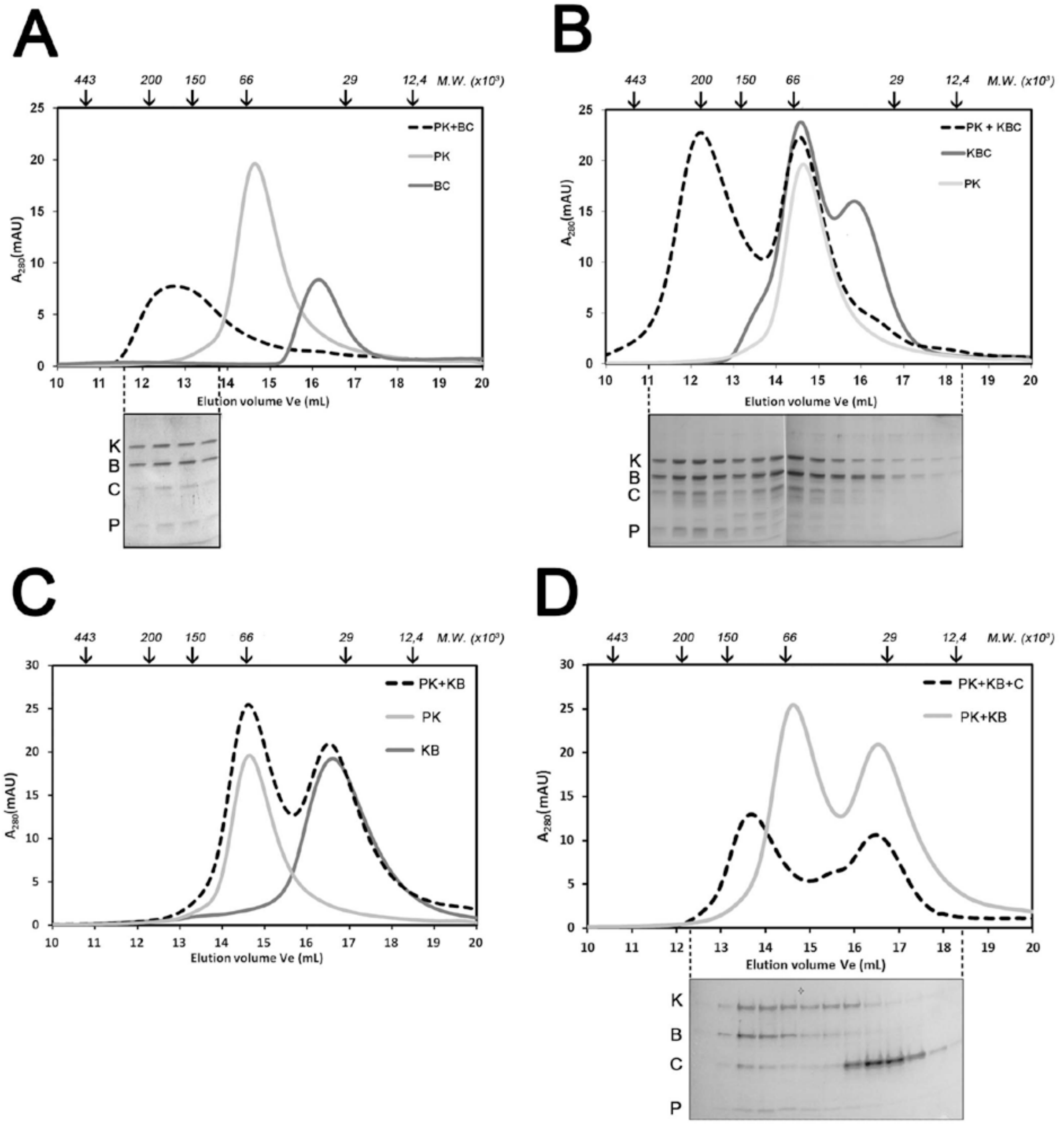

Figure S3. Detection of interactions between PaKEOPS proteins by gel filtration.

Different combinations of subcomplexes and individual subunits were mixed and incubated on ice. The mixtures were analysed by gel filtration and their elution profiles were compared to the ones obtained for individual subcomplexes. Panels A, B and D include the SDS-PAGE analysis of the elution peaks. The molecular masses of the reference proteins used for the calibration of the column are indicated in kDa at the top of each elution profile. The single letter designations are as follows: P - Pcc1, K - Kae1, B – Bud32, C – Cgi121. Elution volumes are indicated on the top of each chromatogram.

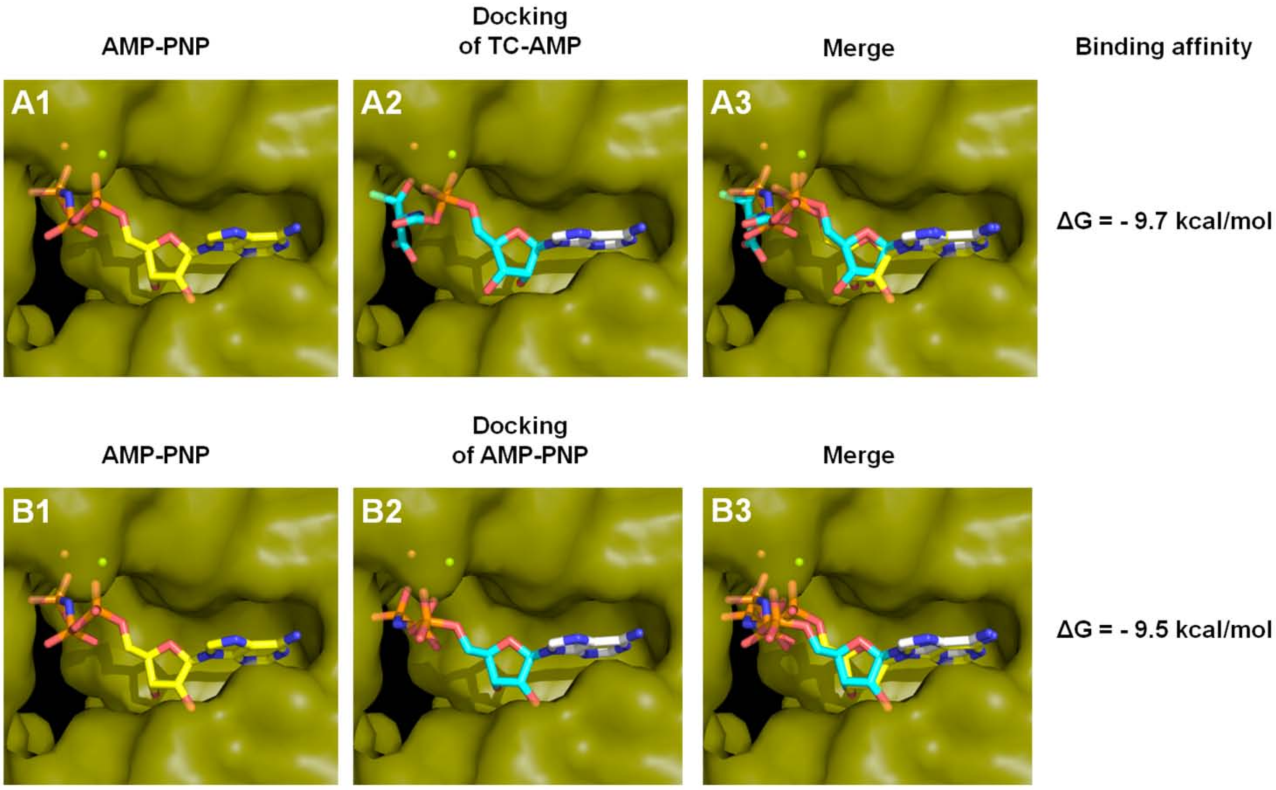

Figure S4. Docking of TC-AMP and AMP-PNP into the nucleotide binding site of Kae1 from *P. abyssi*.

A1 and B1. AMP-PNP bound in the active site cavity of *P. abyssi* Kae1 (PDB file 2IVN). The nucleotide is shown as sticks. A2 and B2. Docking of TC-AMP and AMP-PNP into the active site of *P. abyssi* Kae1. PDB file 2IVN devoid of AMP-PNP was used as template. Blind docking was performed using Autodock Vina 1.1.2 software (see materials and methods). A3 and B3. Superposition of AMP-PNP and docked TC-AMP and AMP-PNP. The calculated molar Gibbs free energy for TC-AMP and AMP-PNP binding is indicated on the right. Fe3+ and Mg2+ ions are shown as orange and red spheres respectively.

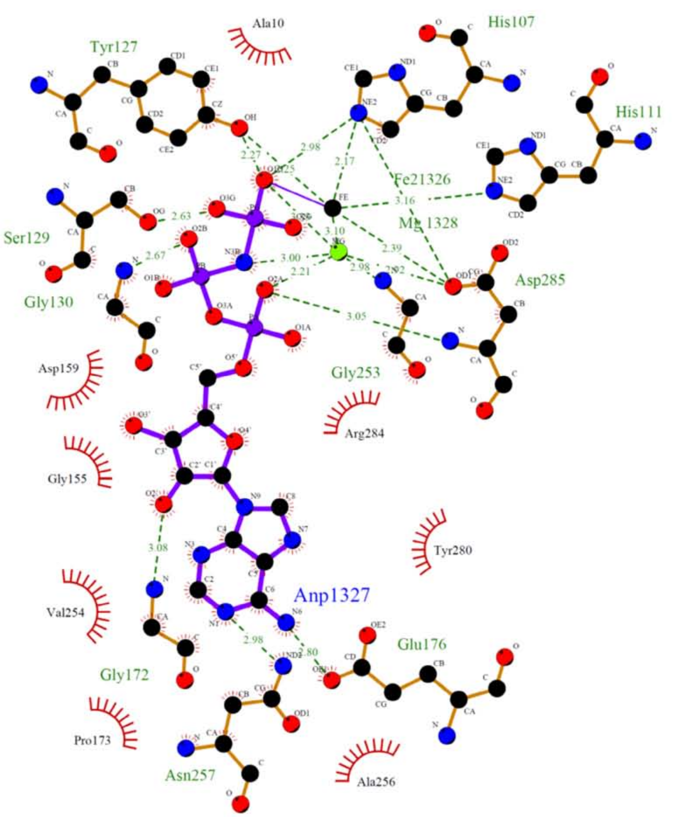

Pa-Kae1 (2ivn)

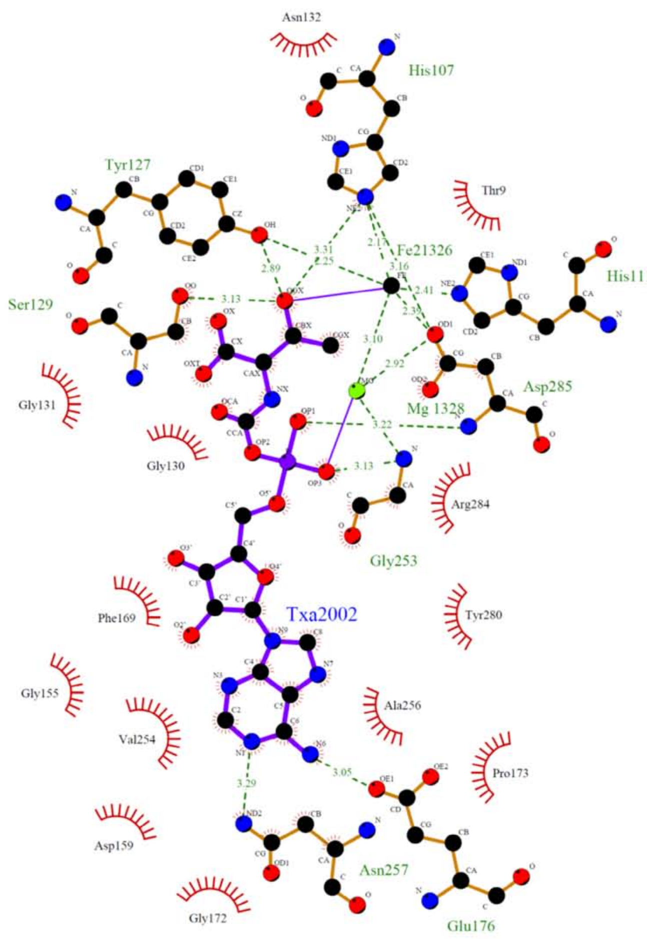

Pa-Kae1 docked with TC-AMP

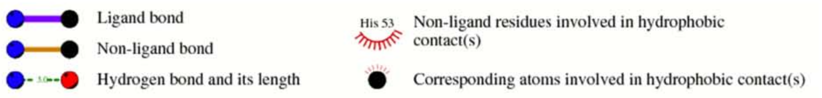

Figure S5. Interaction map for the predicted binding of TC-AMP in the active site of Kae1 from P. abyssi.

Indicated are protein-ligand interactions observed in the co-crystal structure of *P. abyssi* Kae1 and AMP-PNP (2ivn, left panel) and predicted interactions with TC-AMP (right panel). The plots were generated automatically using LigPlot+ v.1.4 (Laskowski and Swindells, 2011).)

References:

Laskowski, RA and Swindells, MB. (2011). LigPlot+: multiple ligand-protein interaction diagrams for drug discovery. *J. Chem. Inf. Model*., **51**, 2778-2786.
